# High glucose macrophage exosomes enhance atherosclerosis by driving cellular proliferation & hematopoiesis

**DOI:** 10.1016/j.isci.2021.102847

**Published:** 2021-07-10

**Authors:** Laura Bouchareychas, Phat Duong, Tuan Anh Phu, Eric Alsop, Bessie Meechoovet, Rebecca Reiman, Martin Ng, Ryo Yamamoto, Hiromitsu Nakauchi, Warren J. Gasper, Kendall Van Keuren-Jensen, Robert L. Raffai

**Affiliations:** 1 Department of Surgery, Division of Vascular and Endovascular Surgery, University of California, San Francisco, San Francisco, CA 94143, USA; 2 Northern California Institute for Research and Education, San Francisco, CA 94121, USA; 3 Neurogenomics, The Translational Genomics Research Institute (TGen), Phoenix, AZ 85004, USA; 4 Institute for Stem Cell Biology and Regenerative Medicine, Stanford University School of Medicine, Lorry I. Lokey Stem Cell Research Building, 265 Campus Drive, Stanford, CA 94305, USA; 5 Department of Genetics, Stanford University School of Medicine, Stanford, CA 94305, USA; 6 Department of Veterans Affairs, Surgical Service (112G), San Francisco VA Medical Center, 4150 Clement St., San Francisco, CA 94121, USA

**Keywords:** Endocrine system physiology, Cell biology

## Abstract

We investigated whether extracellular vesicles (EVs) produced under hyperglycemic conditions could communicate signaling to drive atherosclerosis. We did so by treating Apoe^−/−^ mice with exosomes produced by bone marrow-derived macrophages (BMDM) exposed to high glucose (BMDM–HG-exo) or control. Infusions of BMDM–HG-exo increased hematopoiesis, circulating myeloid cell numbers, and atherosclerotic lesions with an accumulation of macrophage foam and apoptotic cells. Transcriptome-wide analysis of cultured macrophages treated with BMDM–HG-exo or plasma EVs isolated from subjects with type II diabetes revealed a reduced inflammatory state and increased metabolic activity. Furthermore, BMDM–HG-exo induced cell proliferation and reprogrammed energy metabolism by increasing glycolytic activity. Lastly, profiling microRNA in BMDM–HG-exo and plasma EVs from diabetic subjects with advanced atherosclerosis converged on miR-486-5p as commonly enriched and recognized in dysregulated hematopoiesis and *Abca1* control. Together, our findings show that EVs serve to communicate detrimental properties of hyperglycemia to accelerate atherosclerosis in diabetes.

## Highlights

• Macrophages cultured in high glucose (HG) produce pro-atherogenic exosomes
• HG exosomes drive glycolysis in recipient macrophages & monocytosis in Apoe^–/–^ mice
• HG macrophage exosomes & human diabetic PAD plasma EVs are rich in miR-486-5p

## Graphical abstract

**Figure:**
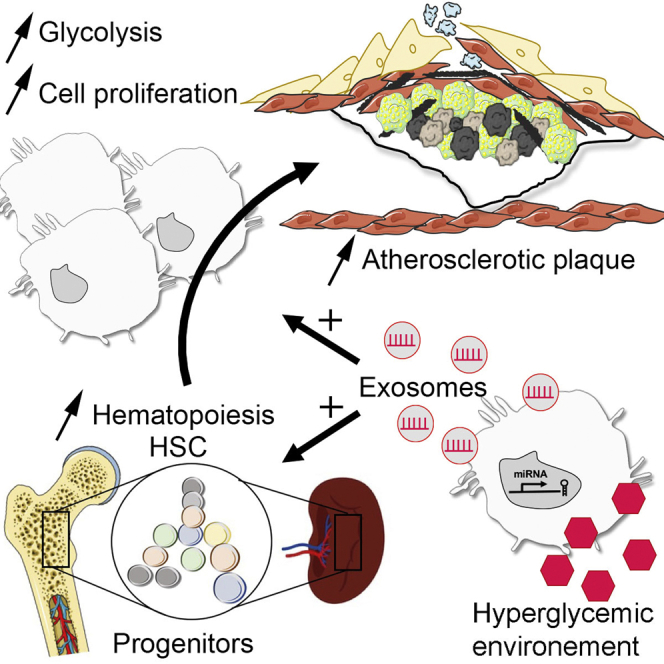

## Introduction

Cardiovascular disease (CVD) is the leading cause of death in the United States, with a predicted increase incidence in the coming decades (Xu et al., 2016). Patients with diabetes mellitus have a substantially elevated risk of coronary, cerebral, and peripheral atherosclerosis where hyperglycemia is the hallmark of the disease (Creager et al., 2003).

Diabetes predisposes to vulnerable atherosclerotic plaques associated with larger foam cell-rich and necrotic core sizes, a greater extent of smooth muscle cell and macrophage apoptosis, and a higher content of inflammatory cell infiltrate compared to nondiabetic atherosclerotic plaques (Burke et al., 2004).

While numerous metabolic disturbances associated with diabetes participate in accelerating atherosclerosis, hyperglycemia is recognized as a central contributor (Creager et al., 2003). Specifically, hyperglycemia is recognized to increase bone marrow (BM) hematopoiesis, and thereby levels of monocytes and neutrophils that readily enter lesions of atherosclerosis prone mice and increase macrophage burden that accelerates atherosclerosis (Flynn et al., 2020; Nagareddy et al., 2013). Lowering blood glucose levels in diabetic mice, including through SGLT2i and insulin mimetic peptide treatment, has recently been shown to reduce BM hematopoiesis and delay atherosclerosis (Kanter et al., 2018; Nagareddy et al., 2013). Collectively, these studies underscore the influence that hyperglycemia exerts on hematopoiesis to drive myelopoiesis and thereby exacerbate atherosclerosis in diabetes. Among cells of the immune system that participate in the process of atherosclerosis, monocytes and macrophages are recognized for their pivotal role in driving accelerated atherosclerosis in diabetes (Kanter et al., 2020; Moreno et al., 2000).

The discovery of extracellular vesicles (EVs) has opened new perspectives in the understanding of complications in diabetes including atherosclerosis. EVs are small bilayer membrane vesicles (30–2000 nm) generated by all cell types and are present in all biological fluids (Mathieu et al., 2019). Exosomes with a size range from 30 to 150 nm are among one type of EVs that have emerged as important mediators of intercellular communication by transporting cargo that include mRNAs, microRNAs, proteins, and lipids (Thery et al., 2009; Valadi et al., 2007; Wen et al., 2018) within local or remote tissues. Numerous studies have now reported on circulating EVs including exosomes with dysregulated microRNA contents among patients with CVD and diabetes, making them useful biomarkers for monitoring disease progression (Freeman et al., 2018; Garcia-Contreras et al., 2017; Lakhter and Sims, 2015; Sorrentino et al., 2020).

Findings from our laboratory recently demonstrated that modulating cultured macrophages toward an anti-inflammatory phenotype fostered the release of exosomes with anti-inflammatory function capable of controlling diet-induced hematopoiesis and atherosclerosis in mice (Bouchareychas et al., 2020). Results of that study support the possibility that exosomes produced by macrophages exposed to metabolic stressors could potentiate atherosclerosis. Thus, our study was designed to investigate cell signaling properties of exosomes produced by macrophages cultured under high glucose conditions.

Our findings show that macrophage exosomes produced in a hyperglycemic environment promote metabolic reprogramming and cellular proliferation that drives hematopoiesis, myelopoiesis, and atherosclerosis in mice. Our data further show that human diabetic plasma EVs profoundly alter transcriptional programs and dysregulated mitochondrial activity when co-cultured with human macrophages. Together, our findings point to exosomes as perpetrators of hyperglycemia in driving the pathogenesis of diabetic atherosclerosis.

## Results

### Assessment of exosomes produced by macrophages cultured in a high glucose environment

In this study, we sought to test the central hypothesis that hyperglycemia causes macrophages to produce exosomes that exert adverse effects in the cardiovascular system. To this end, we cultured BMDM with elevated glucose levels (25 mM glucose) or osmotic normal glucose levels (5.5 mM glucose +19.5 mM mannitol) for 24 h. We verified that the concentration of glucose in the medium remained elevated throughout the 24-h incubation period (Figure S1A).

We then used the dyes CM-H2DCFDA and CellROX to assess ROS production levels in the cells. Our findings shown that culturing BMDM in 25 mM glucose for a period of 24 h substantially increases CM-H2DCFDA levels (Figure S1B) and CellROX signals (Figure S1C). Next, using the MitoSox marker we found that high glucose increases superoxide produced by the mitochondria (Figure S1D). These data confirm that 25mM glucose led to cytosolic and mitochondrial ROS production in cultured primary macrophages. Next, we performed unbiased RNA sequencing analysis of BMDM cultured in high and normal glucose concentrations. We observed that 2,142 genes were differentially regulated by an exposure to high glucose, including 1,196 genes that were upregulated and 946 genes downregulated in high glucose compared to the normoglycemic condition (Figure S1E). Pathway analysis revealed an upregulation of genes implicated in immune responses, oxidative stress, and phagocytosis. Downregulated pathways were generally associated with metabolic processes (Figure S1F).

**Figure 1:**
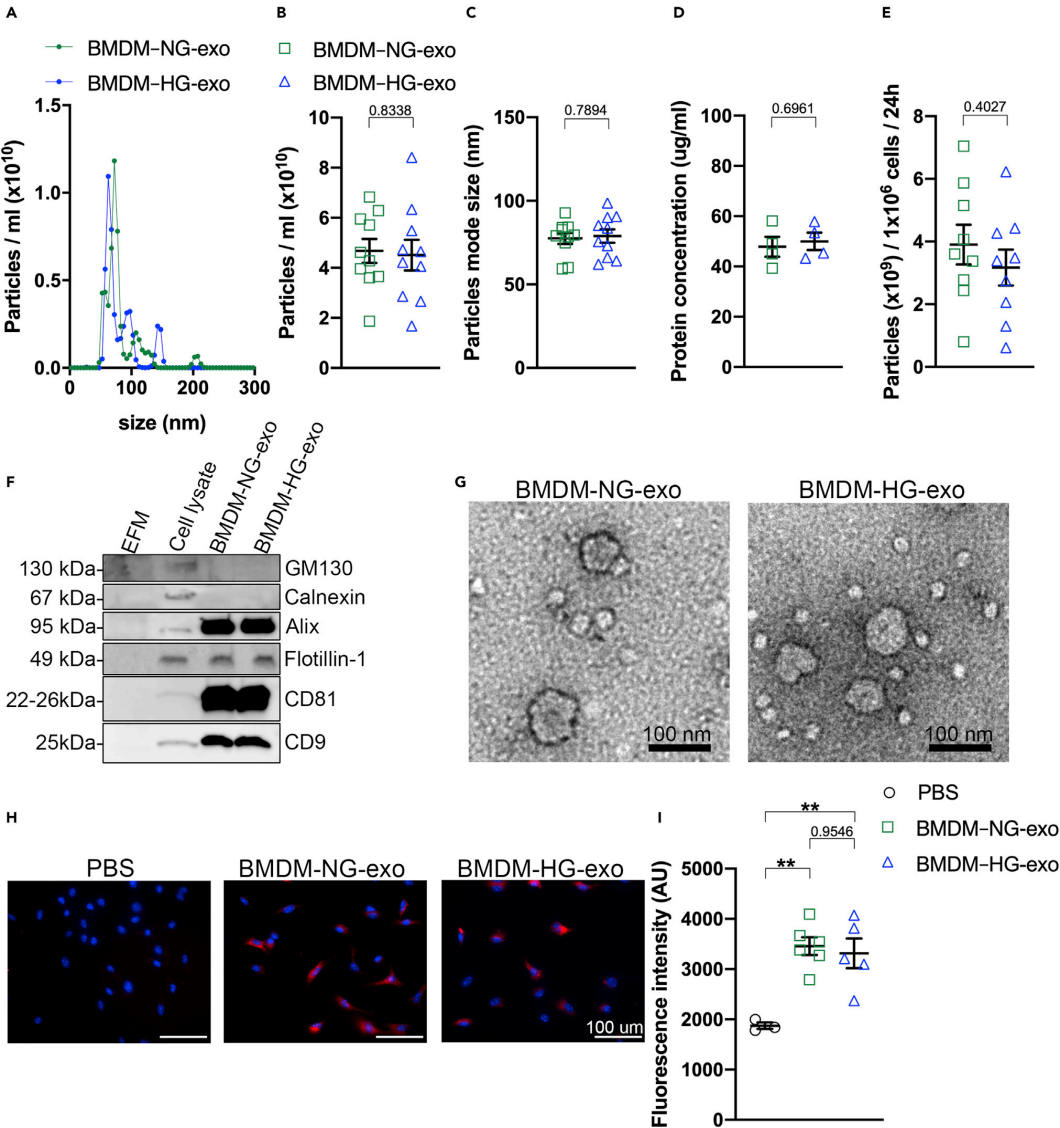
Assessment of exosomes produced by macrophages cultured in a high-glucose environment (A) Representative size and concentration distribution of BMDM–NG-exo or BMDM–HG-exo purified from BMDM conditioned cell culture supernatants after a 24 h period of culture. Measurement of particle concentration (B) and particle mode size (C) using nanoparticle tracking analysis. (D) Quantification of protein concentration by qubit assay. (E) Calculation of secreted particles amounts per million of BMDM in high glucose or low glucose conditions. (F) Western blot analysis of GM130, Calnexin, Alix, Flotillin, and CD81 and CD9 in exosome-free media (EFM), cell lysate, and BMDM–derived exosomes (representative of three independent experiments). An equal volume (37.5 μL) of cDGUC fraction samples was loaded for exosomes analysis and 10 ug of protein was loaded for the cell lysate sample. (G) Electron micrograph of purified exosomes from BMDM cells. Scale bars: 100 nm. (H) Merged images showing internalization of PKH26–labeled BMDM exosomes (red) by naive culture BMDM counterstained with DAPI (blue). BMDM were co-incubated with 2x10^9^ PKH26-labeled exosomes for 2 h at 37°C and washed repeatedly to remove unbound exosomes. All images were acquired using a using a Nikon microscope system with 20× objectives. Scale bars: 100μm. (I) Quantification of the fluorescence intensity.∗p< .05; ∗∗p< .01 as determined by unpaired Student's t test analysis. Data are represented as mean ± SEM.

To examine the impact of metabolic stress in BMDM on their exosomes, we collected them from the conditioned culture medium of BMDM exposed to high concentrations of glucose (BMDM–HG-exo) or normal glucose levels (BMDM–NG-exo) using our recently described C-DGUC approach (Duong et al., 2019). Using NanoSight analysis, we observed a similar particle concentration (Figures 1A and 1B) relatively homogeneous in size (average mode size 77.51 ±3.33 nm for BMDM–NG-exo and 78.92 ±3.99 nm for BMDM–HG-exo) (Figure 1C) that fall within the average range of exosome size. Assessment of protein concentrations of the BMDM exosomes revealed similar levels in both conditions (Figure 1D) and the exosome secretion rate by cells cultured in normoglycemic or hyperglycemic concentration (Figure 1E). We next validated the purity of our exosome preparations by Western blot analysis. We confirmed the absence of non-exosomal markers (GM130 and Calnexin) and the presence of proteins known to be enriched in exosomes (Alix, Flotillin, CD81, and CD9) (Figure 1F). Subsequently, we validated the integrity of the exosomes by transmission electron microscopy (Figure 1G). Lastly, PKH26-labelled BMDM–NG-exo and BMDM–HG-exo were found to be endocytosed at a similar internalization efficiency in recipient BMDM (Figures 1H and 1I).

### BMDM–HG-exosomes increase hematopoiesis and myeloid cell supply

Because hyperglycemia promotes the proliferation and differentiation of hematopoietic stem and progenitor cells (HSPCs) in the BM and spleen, we tested whether BMDM–HG-exo can communicate hyperglycemia-induced hematopoiesis in non-diabetic Apoe^−/−^ mice fed a chow or a Western diet. Cohorts of these Apoe^−/−^ mice were injected intra-peritoneally (IP) with 1 x10^10^ BMDM exosomes or saline (PBS) three times a week for four weeks. We chose an IP over an intravenous (IV) route for exosome infusions as we found this site to be both more practical and similarly effective in delivering exosomes into the circulation after 24 hr (Figure S1A). Importantly, repeated IP exosome treatments did not impact plasma cholesterol levels that remained an average of 445 mg/dL in Apoe^−/−^ mice fed a chow diet (Figure S2B) and 1370 mg/dL for Apoe^−/−^ mice fed a Western diet (Figure S2C).

**Figure 2:**
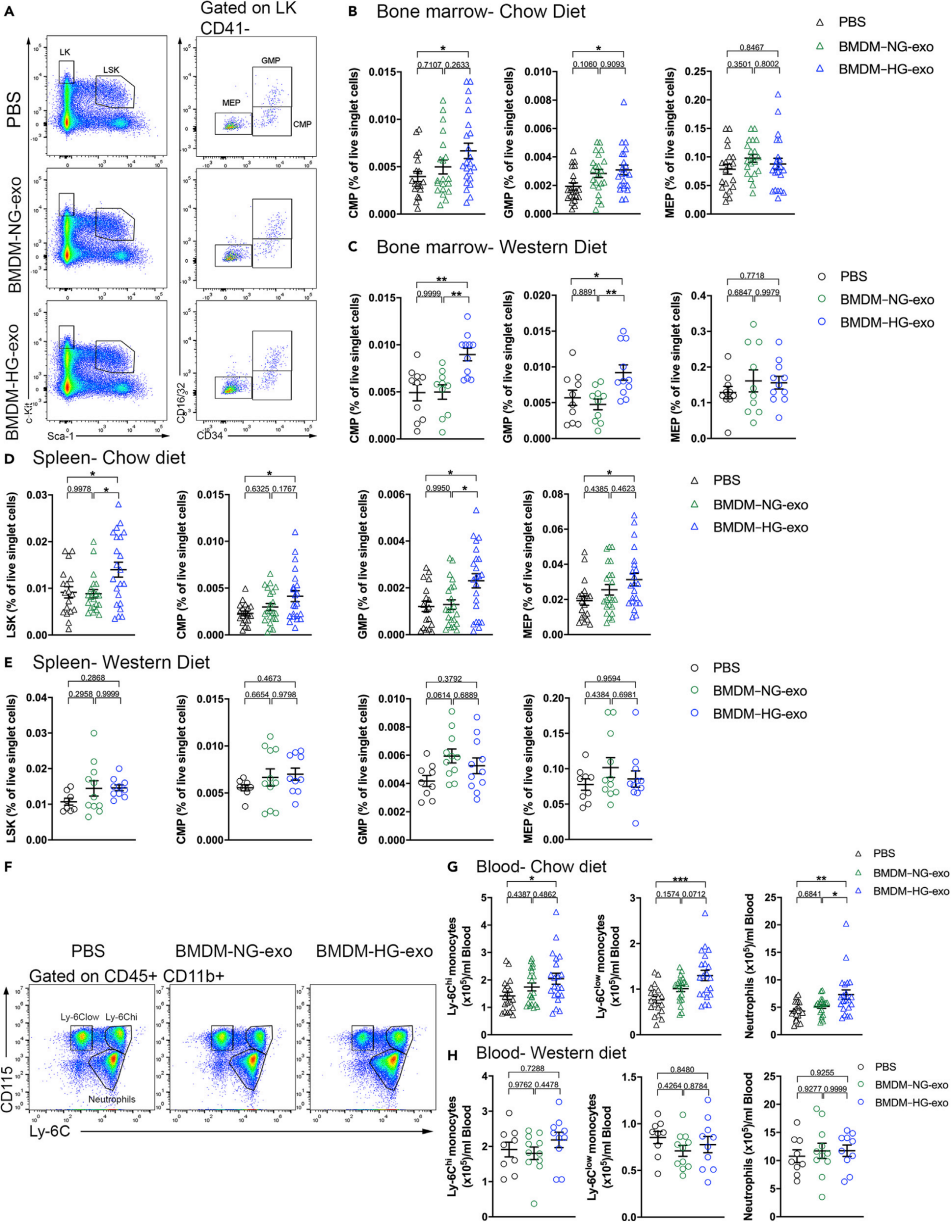
BMDM–HG-exosomes increase hematopoiesis and myeloid cell supply (A) Representative plots of flow cytometric analyses showing the gating strategy for hematopoietic stem and progenitor cells in the BM of Apoe^−/−^ mice fed a chow diet injected with PBS, BMDM–NG-exo or BMDM–HG-exo (1×10^10^ particles/mice every two days) for 4 weeks. LSK cells were defined as Lin– Sca1+ c-Kit+. Common myeloid progenitors (CMPs) were defined as Lin– Sca-1– c-Kit+ (LK) CD41– CD34+ CD16/32–. CMP give rise to granulocyte/macrophage progenitors (GMPs) gated as LK CD41– CD34+ CD16/32+ and megakaryocyte/erythrocyte progenitors (MEPs) defined as LK CD41– CD34– CD16/32–. Doublets and dead cells were excluded prior to analysis. (B) Graph showing the percentage of CMP, GMP, MEP in the BM of Apoe^−/−^ mice fed a chow diet injected with PBS, BMDM–NG-exo or BMDM–HG-exo. A pool of four experiments is shown n = 19–23 mice per group. (C) Graph showing the percentage of CMP, GMP, MEP in the BM of Apoe^−/−^ mice fed a Western diet injected with PBS, BMDM–NG-exo or BMDM–HG-exo. A pool of two experiments is shown n = 10–11 mice per group. (D and E) (D) Analysis of LSK, CMP, GMP, MEP populations in the spleen of Apoe^−/−^ mice fed a chow diet injected with PBS, BMDM–NG-exo or BMDM–HG-exo and (E) Apoe^−/−^ mice fed a Western diet. (F) Representative plots of flow cytometric analyses showing the gating strategy for circulating myeloid cells in Apoe^−/−^ mice fed a chow diet injected with PBS, BMDM–NG-exo or BMDM–HG-exo (1×10^10^ particles/mice every two days) for 4 weeks. (G and H) (G) Quantification of blood monocytes and neutrophils in chow diet fed mice and (H) Western diet fed Apoe^−/−^ mice. Statistical analysis was performed using a two-way ANOVA with Sidak's multiple comparisons post-test. ∗p< 0.05, ∗∗p< 0.01, ∗∗∗p< 0.001. Data are represented as mean ± SEM.

In contrast, we found that injections of BMDM–HG-exo to Apoe^−/−^ fed a chow diet led to marked changes in hematopoiesis as assessed by flow cytometry when compared to mice injected with PBS but not BMDM–NG-exo. Specifically, we detected significant expansions of the common myeloid progenitor (CMP) and granulocyte-macrophage progenitor (GMP) cells (Figures 2A and 2B) in the BM compared to PBS injected control mice.

We detected a similar magnitude in the expansion of the CMP and GMP populations in the BM of Apoe^−/−^ mice fed a Western diet (Figure 2C) after BMDM–HG-exo injections compared to PBS and BMDM–NG-exo injections. However, we noted no changes in megakaryocyte-erythroid progenitor (MEP) populations in either condition.

Turning next to an assessment of splenic hematopoiesis, we noted that repeated injections of BMDM–HG-exo into Apoe^−/−^ fed a chow diet led to robust increases in the LSK, CMP, GMP, and MEP cell populations compared to control injected mice (Figure 2D). In contrast, such marked expansion of progenitor cell populations caused by BMDM–HG-exo were not apparent in Apoe^−/−^ mice fed a Western diet (Figure 2E). Similarly, an assessment of circulating leukocytes by flow cytometry revealed that repeated injections of BMDM–HG-exo compared to PBS and BMDM–NG-exo led to an increase among circulating Ly-6C^low^ monocytes and neutrophils only in Apoe^−/−^ mice fed a chow diet (Figures 2F and 2G). An analysis of myeloid cell populations in Apoe^−/−^ mice fed a Western diet showed no differences in monocyte subtypes and neutrophils after repeated injections of BMDM exosomes (Figure 2H), which could in part be due to overwhelming proliferative signaling caused by hyperlipidemia in this model (Swirski et al., 2007; Yvan-Charvet et al., 2010). Taken together, our findings revealed that BMDM–HG-exo can modulate both BM and extramedullary hematopoiesis to augment leukocyte supply in the circulation of Apoe^−/−^ mice fed a chow diet.

### BMDM–HG-exo accelerate spontaneous and diet-induced atherosclerosis in Apoe^−/−^ mice

**Figure 3:**
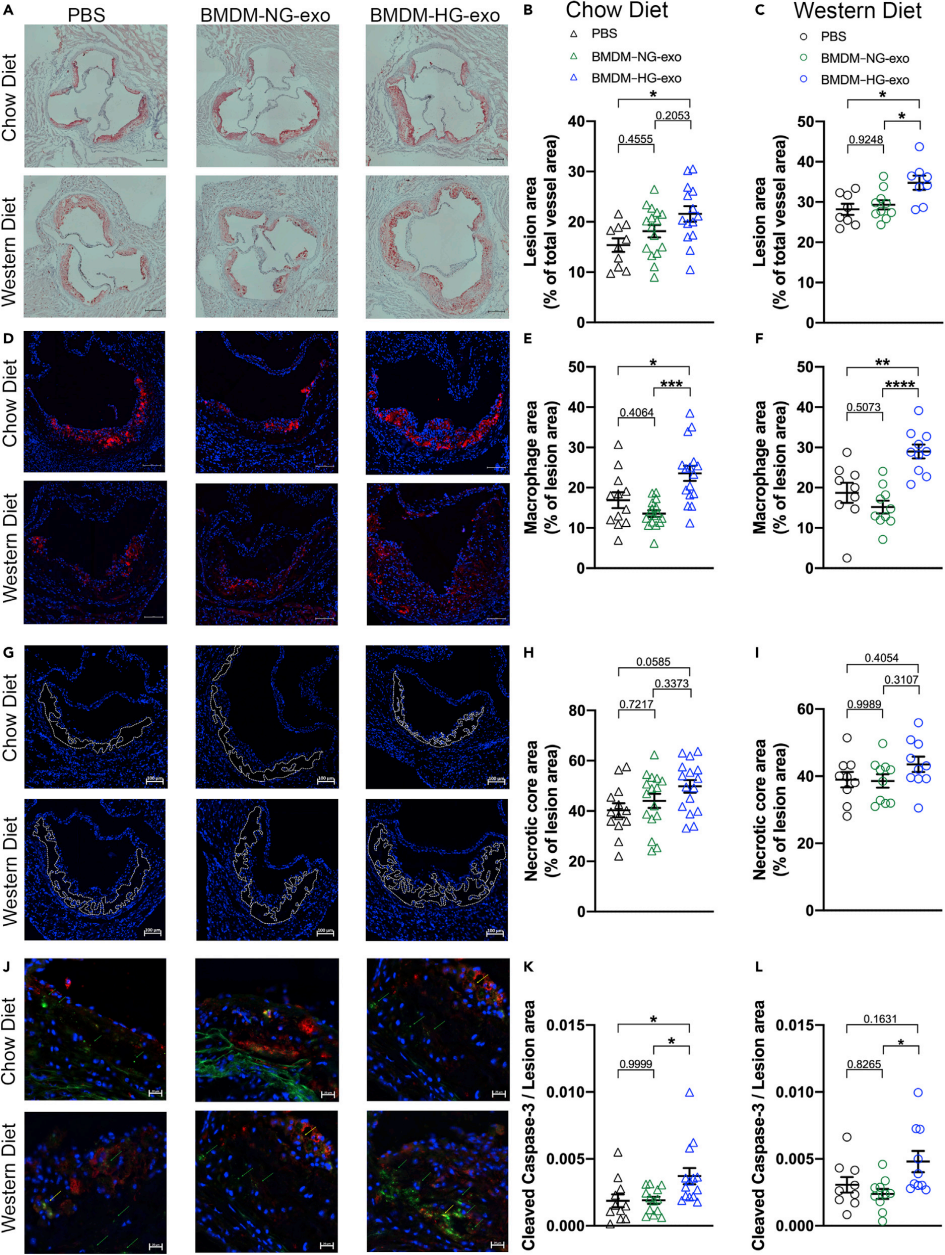
BMDM–HG-exo accelerate spontaneous and diet-induced atherosclerosis in Apoe^−/−^ mice Apoe^−/−^ mice were subjected to PBS, BMDM–NG-exo or BMDM–HG-exo (1×10^10^ particles/mice every two days) for 4 weeks. (A and B) (A) Representative images and (B) quantification of Oil Red O (ORO) staining in Apoe^−/−^ mice fed a chow diet. Pool of four independent experiments is shown, n = 10–16 mice in each group. Scale bars: 200μm. (C) Quantification of ORO staining in Apoe^−/−^ mice fed a Western diet. Pool of two independent experiments is shown, n = 8–10 mice in each group. (D) Representative images of MOMA-2^+^ macrophages in the atherosclerotic plaques of aortic root areas. Scale bars: 100μm. (E) Macrophage content analysis via immunodetection of MOMA-2 in Apoe^−/−^ mice fed a chow diet. Pool of four independent experiments is shown, n = 12–16 mice in each group. (F) Macrophage content analysis in Apoe^−/−^ mice fed a Western diet. Pool of two independent experiments is shown, n = 9–10 mice in each group. (G) Representative cross-sectional view of aortic root stained with DAPI to measure necrosis area from each group of mice. Dashed lines show the boundary of the developing necrotic core. Scale bars: 100 μm. (H) Quantification of necrotic core area as a percent of total plaque area in Apoe^−/−^ mice fed a chow diet. Pool of four independent experiments is shown, n = 13–16 mice in each group. (I) Quantification of necrotic core area in Apoe^−/−^ mice fed a Western diet. Pool of two independent experiments is shown, n = 9–10 mice in each group. (J) Representative image of cleaved caspase-3 and MOMA-2 staining in aortic root lesions. Scale bars: 20 μm. (K) Quantification of cleaved caspase-3 staining as a ratio of lesion area in Apoe^−/−^ mice fed a chow diet. Pool of four independent experiments is shown, n = 11–14 mice in each group. (L) Quantification of cleaved caspase-3 in Apoe^−/−^ mice fed a Western diet. Pool of two independent experiments is shown, n = 9–10 mice in each group. Statistical analysis was performed using one-way ANOVA and Sidak's multiple comparisons post test. ∗p< 0.05, ∗∗p< 0.01, ∗∗∗p< 0.001, ∗∗∗∗p< 0.0001. Data are represented as mean ± SEM.

As increased myelopoiesis is recognized to contribute to atherosclerotic plaque progression (Murphy et al., 2011; Robbins et al., 2012; Swirski et al., 2007), we tested whether BMDM–HG-exo can impact the course of atherogenesis in Apoe^−/−^ mice fed a chow or Western diet. We did so through injections at a dose of 1 x10^10^ particles, three times a week for 4 weeks, a dose of exosomes that represent 4.5% of normal circulating exosomes levels (Huang et al., 2018). Interestingly, while injections of BMDM–HG-exo caused increased myelopoiesis only in chow-fed Apoe^−/−^ mice, they accelerated atherosclerosis in the aortic root of both groups of mice compared to mice injected with controls (Figures 3A–3C). This acceleration of atherosclerosis by BMDM–HG-exo occurred in part by an increase in the number of lesional macrophages as detected by MOMA-positive immunofluorescent staining in Apoe^−/−^ fed a chow diet (Figures 3D and 3E) and in Apoe^−/−^ fed a Western diet (Figure 3F) as compared to mice treated with saline or BMDM–NG-exo. In contrast, measurements of the necrotic core area (Figure 3G) showed no significant impact of the BMDM-exo both in chow (Figure 3H) and Western diet fed (Figure 3I) Apoe^−/−^ mice. However, when looking at cellular apoptotic events in the lesions by cleaved caspase 3 staining (Figure 3J), we noted markedly increased staining in lesions of Apoe^−/−^ fed a chow diet injected with BMDM–HG-exo compared to controls injected mice (Figure 3K). Quantification of the apoptotic cell content in Western diet fed mice while increased, did not reach statistical significance (Figure 3L). Altogether, findings from our study show that BMDM–HG-exo enhance atherosclerosis by increasing lesional macrophage content and apoptotic cell numbers, two morphological features that are frequently observed in atherosclerotic plaques among human diabetic subjects (Burke et al., 2004).

### Assessment of plasma EVs isolated from subjects with diabetes and atherosclerosis

**Figure 4:**
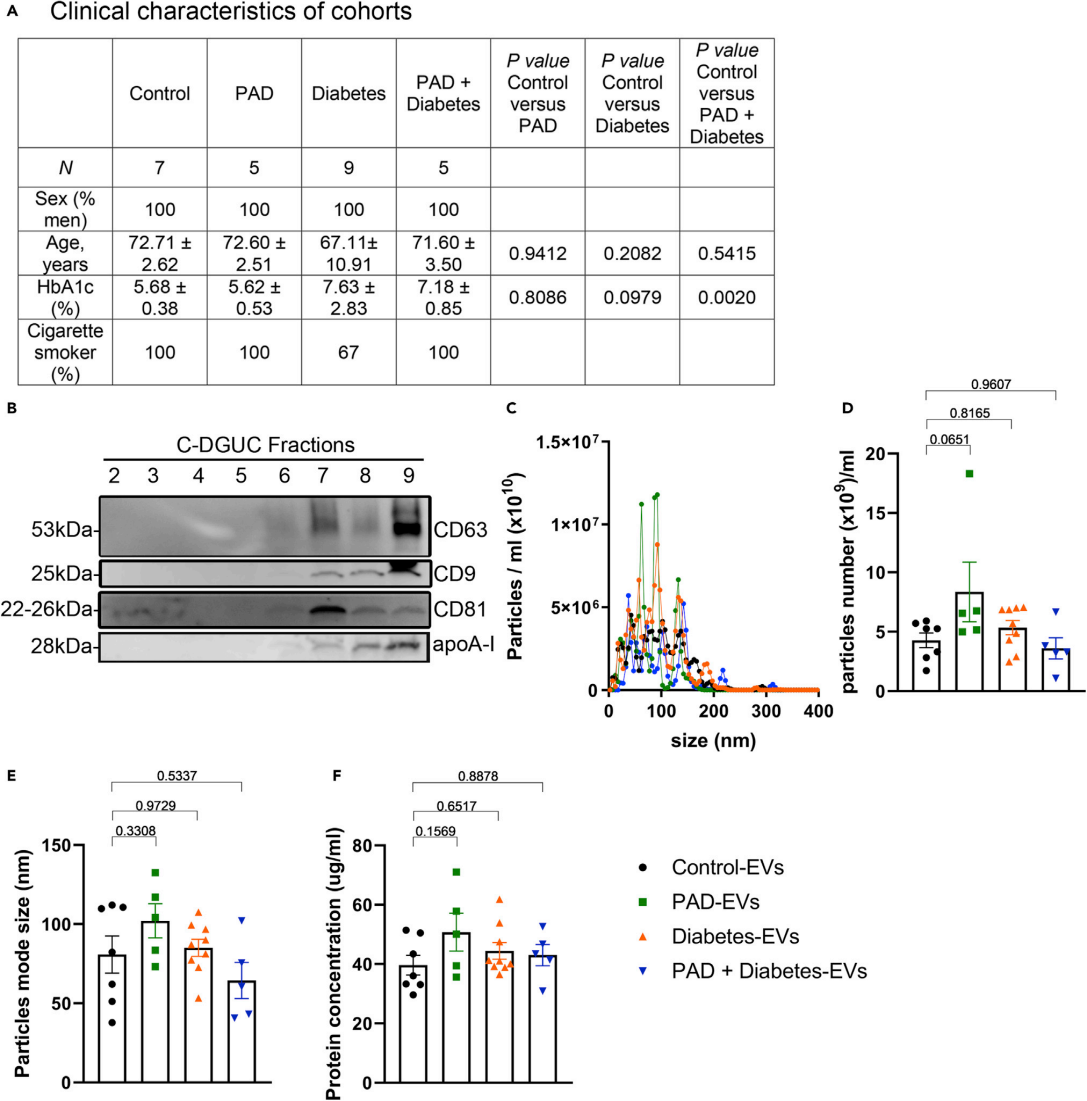
Assessment of plasma EVs isolated from subjects with diabetes and atherosclerosis (A) Details of clinical data of the cohort used in this study. (B) Analyses of 9 individual fractions of plasma EVs separated by C-DGUC method. An equal volume (37.5 μL) of fraction samples was loaded for Western blot analysis. (C–E) (C) Particle diameter distribution profiles for fraction 8 estimated by NTA. Particle concentrations (D) and size (E) were estimated by NTA using NanoSight. (F) Protein concentration was measured by qubit assay. Statistical analysis was performed using one-way ANOVA and Sidak's multiple comparisons post-test. ∗p< 0.05, ∗∗p< 0.01, ∗∗∗p< 0.001, ∗∗∗∗p< 0.0001. Data are represented as mean ± SEM.

In beginning to test the human relevance of exosomes produced under hyperglycemic conditions, we isolated circulating exosomes from 4 different groups of subjects: Healthy (Control-EVs), patients with peripheral arterial disease (PAD-EVs), patients with diabetes and no known coronary or peripheral artery disease (Diabetes-EVs) or diabetes associated with PAD (PAD + Diabetes-EVs) (Figure 4A). We used the terminology EVs to refer to human exosomes as there is more vesicle heterogeneity in particle sizes and contamination of other vesicular structures in human EVs as compared to EVs isolated from cultured cells. Plasma EVs were isolated using our C-DGUC method (Duong et al., 2019), the same method used to isolate BMDM-exosomes from conditioned culture medium.

To determine the fraction most enriched with EVs, we performed a Western blot analysis of the 12 collected fractions for the detection of EV-associated markers, CD63, CD9, and CD81 (Figure 4B). We observed that fractions 7 and 8 are the ones most enriched in exosomes with minimal plasma protein contaminants such as ApoA-I, and therefore these 2 fractions served for subsequent experiments.

NanoSight analysis of fraction 8 revealed a similar profile of EVs between all groups (Figure 4C) with particles ranging in sizes and a similar particle concentration per ml of plasma (Figure 4D). For the Control-EVs, we observed a mode size of 80 ± 11.74 nm, 102.1 ± 10.79 nm for PAD-EVs, 85.07 ± 5.41 nm for Diabetes-EVs and 64.44 ± 11.36 nm for PAD + Diabetes-EVs (Figure 4E). The measured protein concentration was similar between all groups (Figure 4F).

### Diabetic plasma EVs communicate molecular changes in recipient macrophages

**Figure 5:**
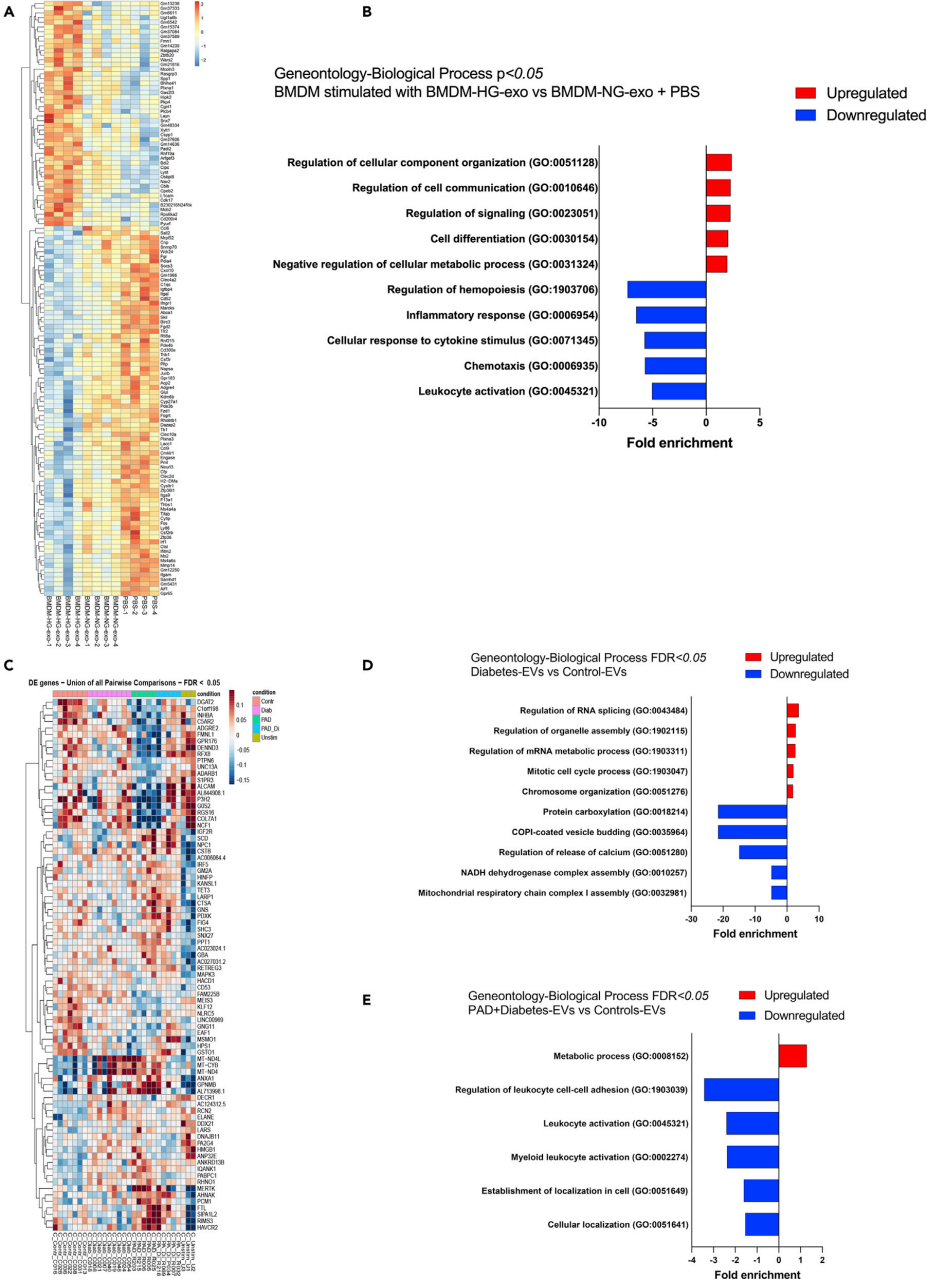
EVs induce molecular changes in macrophage recipient cells (A) Heatmap showing the distinct mRNA expression profiles between BMDM exposed to BMDM–HG-exo (n = 4) versus controls (PBS (n = 4) combined with BMDM–NG-exo (n = 4)) for 24 h, analyzed with log2FC cut-off to 0.15. Each sample were from separate BMDM-exo preparations. (B) GO enrichment analysis (Biological process) of the gene differentially expressed between BMDM exposed to BMDM–HG-exo and controls (BMDM–NG-exo + PBS). The minimum count of genes considered for the analysis was >10 and p <0.05. (C) Heatmap displaying all differentially expressed genes and their normalized read counts in THP-1 cells treated with Healthy (Control-EVs, n = 7), Diab (Diabetes-EVs, n = 9), PAD (PAD-EVs, n = 5), PAD_Di (PAD + Diabetes-EVs, n = 5), and PBS stimulated cells (Unstim, n = 3) for 24 hr (D and E) (D) GO enrichment analysis (Biological process) of the gene differential expressed (p<0.05) between Diabetes-EVs and Control-EVs with (E) GO enrichment analysis (Biological process) of the gene differential expressed (p<0.05) between PAD +Diabetes-EVs and Control-EVs. The minimum count of genes considered for the analysis was >6.

To explore biological functions of mouse macrophage exosomes and human diabetic plasma EVs *in vitro*, we performed RNA sequencing analysis of transcripts altered in recipient cells. Specifically, we tested naive murine BMDM exposed to PBS, BMDM–NG-exo and BMDM–HG-exo, as well as THP-1 cells exposed to human plasma EVs. Differential expression analysis revealed an upregulation of 57 genes and downregulation of 89 genes in BMDM exposed to BMDM–HG-exo compared to combined BMDM–NG-exo and PBS treated cells (Figure 5A). Furthermore, we noted that genes involved in pathways associated with cellular component organization and cell differentiation were upregulated, while hematopoiesis and immune response pathways were downregulated by BMDM–HG-exo (Figure 5B). We also noted an upregulation of *Bcl2* expression in BMDM stimulated with BMDM–HG-exo, a key regulator of cellular apoptosis and critical modulator of HSC survival (Domen et al., 2000) and cellularity in atheroma (Bouchareychas et al., 2015). We also noted that BMDM–HG-exo reduced the expression of *Abca*1 that is central to promoting cholesterol efflux from macrophages and HSPC preventing their expansion and contribution to atherosclerosis (Yvan-Charvet et al., 2010). RNA Seq analysis of THP-1 cells exposed to circulating human EVs revealed differential expression of genes depending on the source of EVs (Figure 5C). EVs isolated from the plasma of diabetic subjects upregulated the expression of genes implicated in cellular proliferation and revealed a downregulation of genes implicated in the OXPHOS energy metabolism pathway and mitochondrial respiratory electron transport and ATP synthesis (Figure 5D) suggesting their capacity to disrupt mitochondrial complex function. Pathway analysis of PAD + Diabetes-EVs compared to Control-EVs revealed an upregulation of genes implicated in metabolic process and downregulation of genes implicated in leukocyte activation and cellular localization (Figure 5E). Together, our findings reveal that EVs produced in high glucose environments can substantially impact the expression of genes involved in metabolic and inflammatory pathway in recipient macrophages.

### BMDM–HG-exo modulate macrophage energy metabolism

**Figure 6:**
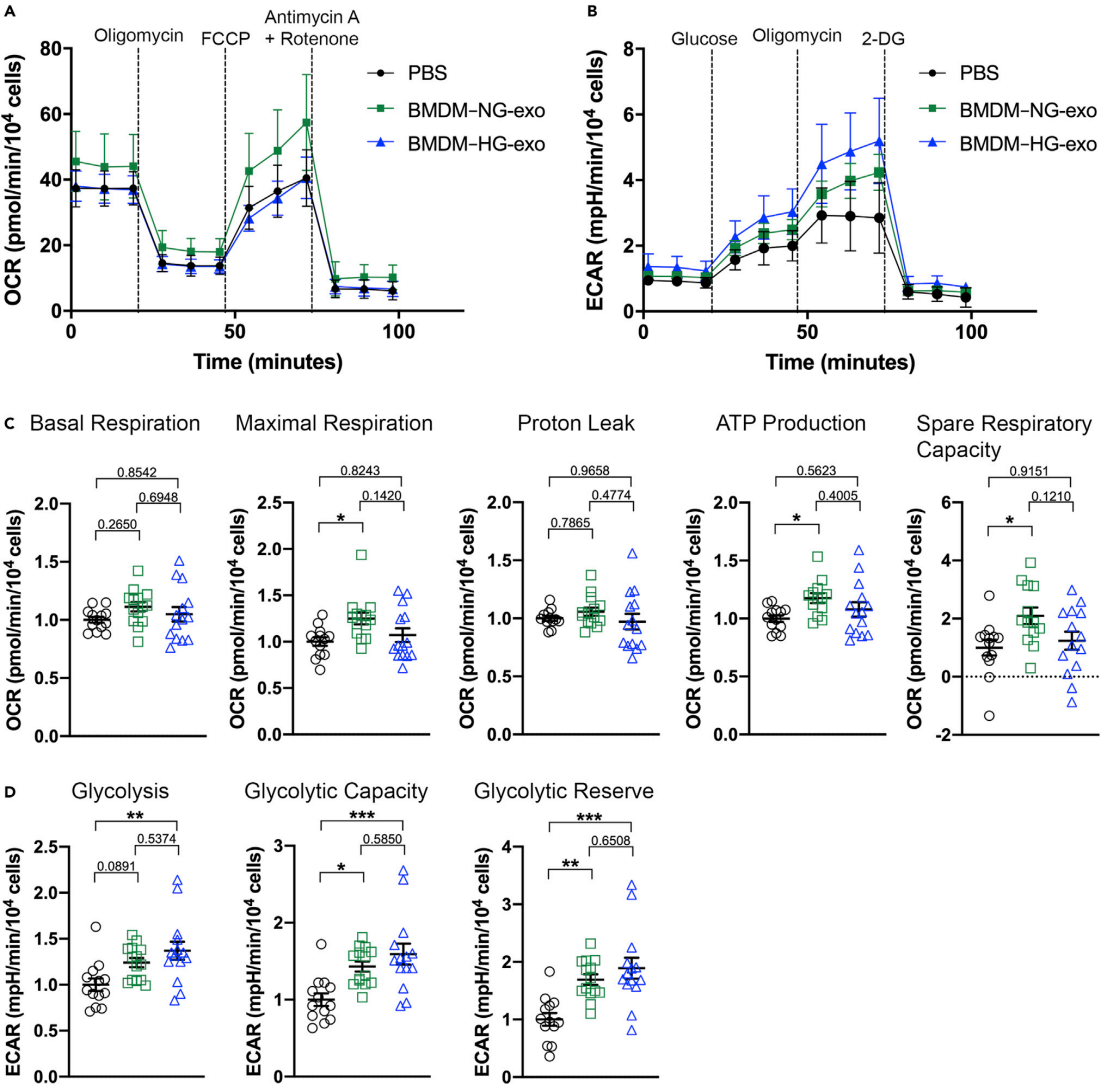
BMDM–HG-exo modulate macrophage energy metabolism (A and B) (A) Graph showing representative seahorse extracellular flux analysis of oxygen consumption rate (OCR) and (B) extracellular acidification rate (ECAR) in BMDM exposed to PBS, BMDM–NG-exo or BMDM–HG-exo for 24 hr. One representative experiment out of two experiments is shown n = 13–14 per group). (C) Bar graphs showing quantified cell-normalized mitochondrial OCR from stress tests. Results are presented relative to PBS control. (D) Bar graphs showing quantified cell-normalized glycolytic pathway. Results from a pool of two independent experiments is shown, n = 12–14 in each group. ∗p< 0.05, ∗∗p< 0.01, ∗∗∗p< 0.001 as determined by one-way ANOVA and Holm-Sidak post test. Data are represented as mean ± SEM.

In following up on these observations, we examined whether BMDM–HG-exo can influence macrophage energy metabolism as we reported for exosomes produced by macrophages exposed IL-4 (Bouchareychas et al., 2020). First, we determined the oxygen consumption rate (OCR) including basal and maximal respiration, proton leak, ATP production and spare respiratory capacity in BMDM stimulated with PBS, BMDM–NG-exo or BMDM–HG-exo (Figure 6A). We observed an increase of maximal respiration, and spare respiratory capacity in cells exposed to BMDM–NG-exo compared to PBS control. However, we also observed that BMDM–HG-exo failed to increase OxPhos in recipient BMDM (Figure 6C).

Next, we tested the effect of BMDM–HG-exo on modulating the extracellular acidification rate (ECAR), an indicator of aerobic glycolysis (Figure 6B). Glycolysis rate, glycolytic capacity and glycolytic reserve were all significantly higher in macrophages stimulated with BMDM–HG-exo compared to PBS treated cells (Figure 6D). Of note, cells stimulated with BMDM–NG-exo also showed an increased glycolytic capacity and glycolytic reserve, but these changes were more notable in cells treated with BMDM–HG-exo.

Collectively, these findings demonstrate that BMDM–NG-exo upregulated both mitochondrial function and glycolysis to obtain the ATP necessary for their survival. However, BMDM–HG-exopromoted a metabolic switch among recipient cells toward a preferential use of glucose for energy production.

### BMDM–HG-exo modulate cell proliferation in macrophages and myeloid progenitors

**Figure 7:**
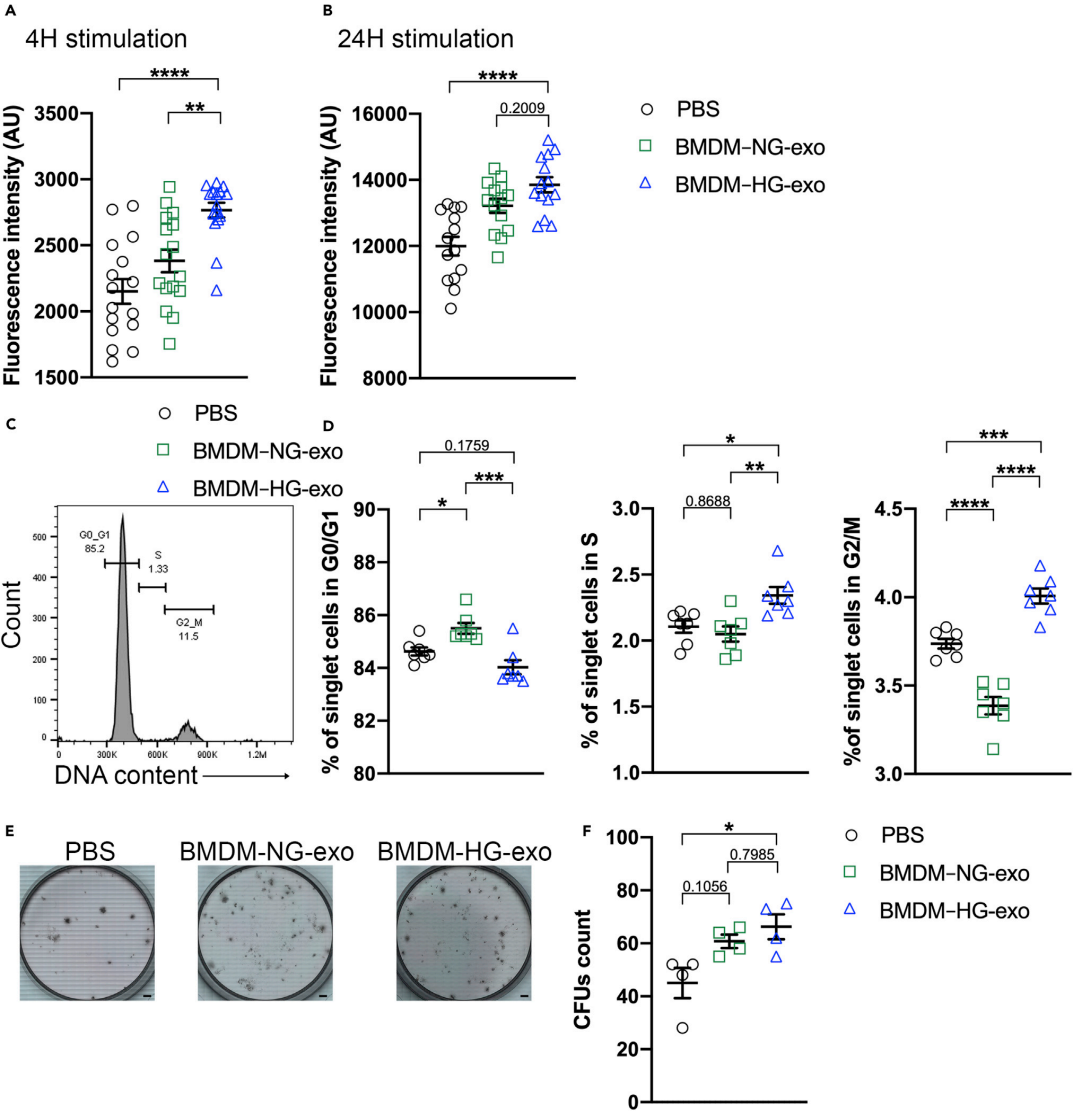
BMDM–HG-exo modulate cell proliferation in macrophages and myeloid progenitors (A and B) Measurement of fluorescent AlamarBlue signal in BMDM exposed to PBS, BMDM–NG-exo or BMDM–HG-exo during (A) 4 hr or (B) 24 hr. (C) Flow cytometry histogram showing the gating strategy for the measurement of cell cycle distribution in BMDM after treatment with PBS, BMDM–NG-exo or BMDM–HG-exo for 4 hr. (D) Quantitative analysis of cell cycle distribution. One representative experiment out of two experiments is shown. (E and F) (E) Representative images of CFU assay (F) 2×10^4^ BM cells from C57BL/6 mice were plated in Methylcellulose-based medium with recombinant cytokines for colony-forming unit (CFU) and treated every two days with PBS, BMDM–NG-exo or BMDM–HG-exo at a dose of 2×10^9^ particles/ml. Pool of four experiments with 4 separate preparations of exosome were analyzed. Scale bars: 4.37mm. Statistical analysis was performed using a two-way ANOVA with Sidak's multiple comparisons post-test. ∗p< 0.05, ∗∗p< 0.01, ∗∗∗p< 0.001, ∗∗∗∗p< 0.0001. Data are represented as mean ± SEM.

Having established their ability to modulate cellular energy metabolism, we next tested whether BMDM exosomes could impact cell proliferation. We performed an AlamarBlue assay with naive BMDM exposed to PBS, BMDM–NG-exo or BMDM–HG-exo and observed an increase in fluorescent signal demonstrating an increase in cell viability and therefore proliferation after 4 hr (Figure 7A) and 24 hr (Figure 7B) of BMDM–HG-exo treatment. Next, we measured cellular DNA content to detect the cell cycle distribution after treatment with PBS, BMDM–NG-exo or BMDM–HG-exo (Figure 7C). Our data consistently revealed an increase in the percentage of cells in phase S and G2/M (Figure 7D) among BMDM stimulated with BMDM–HG-exo compared to BMDM–NG-exo and PBS. These findings show that BMDM–HG-exo can serve to stimulate the proliferation of cultured macrophages. Next, we tested the impact that BMDM–HG-exo could exert to support hematopoietic stem cell function using a colony forming assay (CFU) assay (Figure 7E). Freshly isolated BM cells exposed to BMDM–HG-exo displayed increased colony numbers compared to those exposed to PBS but not BMDM–NG-exo (Figure 7F) indicating that BMDM–HG-exo have a capacity to further enhance hematopoietic stem cell proliferation. Altogether, these data indicate that BMDM–HG-exo display a capacity to promote the proliferation of cells central to the pathogenesis of atherosclerosis.

### Hyperglycemia dysregulates microRNA in myeloid cells and their EVs

Numerous studies have reported changes in circulating microRNA levels associated with accelerated atherosclerotic disease in diabetes (de Candia et al., 2017; Zampetaki et al., 2010). As we and others (Gaudreault et al., 2013; Nagareddy et al., 2013) observed that myeloid cells are impacted by hyperglycemia, we performed microRNA sequencing on Ly-6C^hi^ monocytes and macrophages isolated from mouse models of hyperglycemia that included the STZ-induced and spontaneous Akita mouse models of diabetes.

Our findings revealed differential expression of 23 microRNA in Ly-6C^hi^ monocytes sorted from the blood of STZ-treated mice compared to controls (Figure S3A). In the Akita mouse model, only 4 microRNA were differentially expressed in the Ly-6C^hi^ monocytes compared to control with miR-486-5p found significantly upregulated (Figure S3B). In peritoneal macrophages isolated from the Akita mouse model, we also observed an upregulation of 24 microRNA and downregulation of 14 microRNA relative to wild-type mice (Figure S3C). Next, we analyzed microRNA expression profiles of BMDM exposed to normal or high glucose concentration. We observed a downregulation of 11 microRNA and an upregulation of 22 microRNA (Figure S3D).

**Figure 8:**
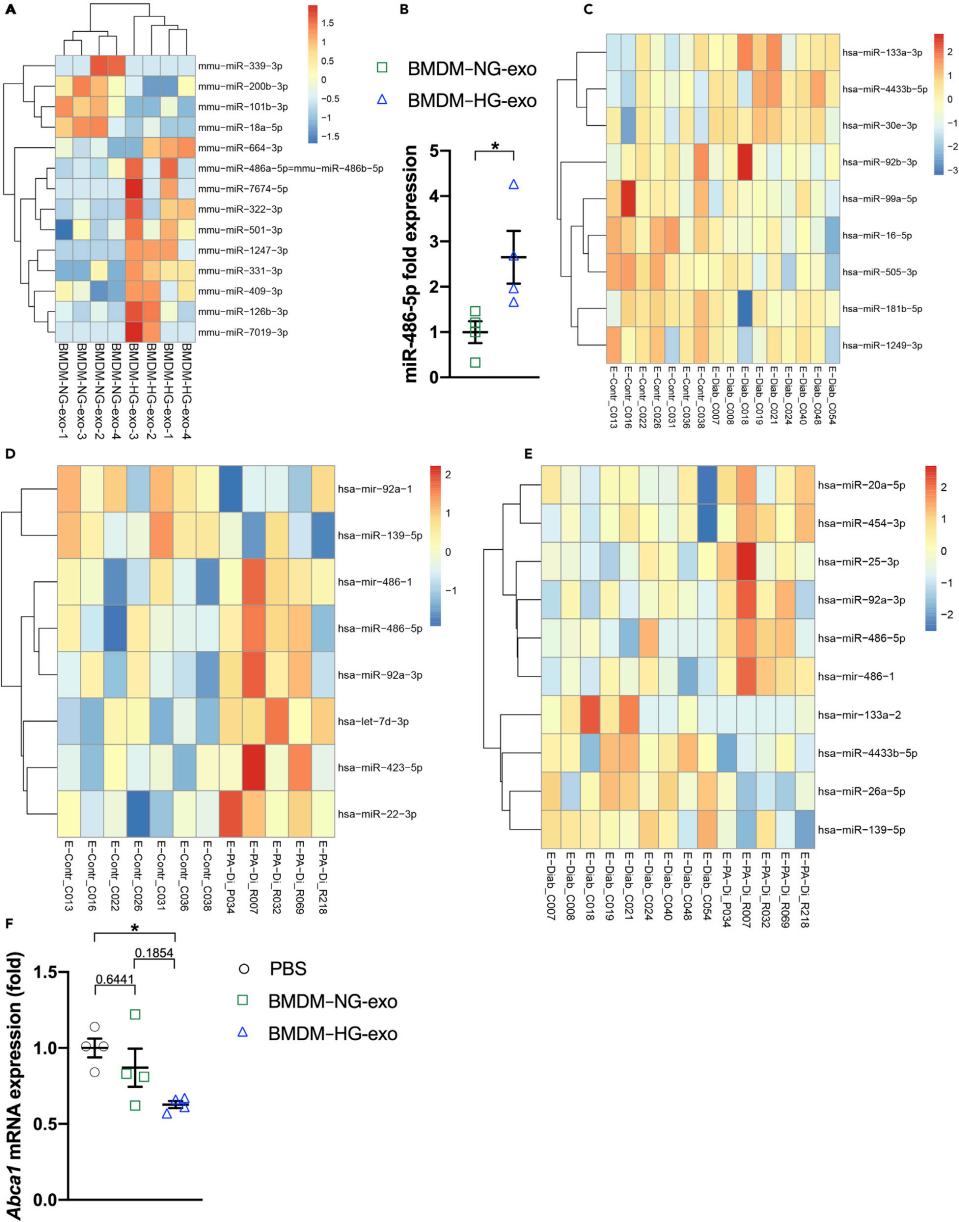
Hyperglycemia dysregulates microRNA in myeloid cells and their EVs (A) Heatmap showing the distinct microRNA expression profiles between BMDM–NG-exo (n = 4) and BMDM–HG-exo (n = 4) (p <0.05). (B) qRT-PCR analysis of miR-486a-5p in BMDM–NG-exo and BMDM–HG-exo. Each sample were from separate BMDM-exo preparations. ∗p < .05 as determined by unpaired Student's t test analysis. (C) Heatmap illustrating microRNA differential expression in the circulating EVs of healthy subjects (Control-EVs, n = 7) compared to EVs isolated from diabetic patients (Diabetes-EVs, n = 9). (D) Heatmap illustrating microRNA differential expression in the circulating EVs of healthy subjects (Control-EVs, n = 7) compared to EVs isolated from patients with diabetes and PAD (PAD + Diabetes-EVs, n = 5). (E) Heatmap illustrating microRNA differential expression in the circulating EVs isolated from diabetic patients (Diabetes-EVs, n = 9) or patients with diabetes and PAD (PAD + Diabetes-EVs, n = 5). Red signal and blue signal indicate microRNA expression levels. (F) qRT-PCR analysis of *Abca1* mRNA expression in BMDM after treatment with PBS, BMDM–NG-exo or BMDM–HG-exo for 24 hr. One representative experiment out of two experiments is shown n = 4 per group. Statistical analysis was performed using a two-way ANOVA with Sidak's multiple comparisons post-test. ∗p< 0.05. Data are represented as mean ± SEM.

A comparison of shared microRNA changes in peritoneal macrophages isolated from Akita mice and BMDM–HG cell population revealed an increase in levels of miR-126a-3p, miR-99b-5p, miR-16-5p and miR-423-3p in both hyperglycemic models. Next, we performed small RNA sequencing on both BMDM exosomes and human circulating EVs isolated from control and diabetic patients including those with established vascular disease. As shown Figure 8A, microRNA sequencing revealed 14 differentially expressed microRNA in BMDM–HG-exo compared to BMDM–NG-exo including 10 upregulated and 4 downregulated microRNA. Interestingly we observed that miR-486-5p was enriched in BMDM–HG-exo that parallel their increased expression in myeloid cells examined from hyperglycemic mouse models (Figure S3B). The increased miR-486-5p levels in BMDM–HG-exo compared to BMDM–NG-exo were further validated and confirmed using qRT-PCR (Figure 8B).

An analysis of microRNA levels in human circulating EVs showed few differences between Control-EVs and Diabetes-EVs (Figure 8C). However, we noted that PAD + Diabetes-EVs displayed 6 upregulated and 2 downregulated microRNA compared to Control-EVs (Figure 8D). Furthermore, some of these microRNA were also noted to be upregulated when comparing PAD + Diabetes-EVs and Diabetes-EVs (Figure 8E). Specifically, we noted that miR-486-5p was upregulated in PAD + Diabetes-EVs compared to Diabetes-EVs and Control-EVs. Furthermore, miR-486-5p was found to be upregulated in BMDM–HG-exo compared to BMDM–NG-exo (Figure 8A). Because miR-486-5p has been shown to reduce the expression of ABCA1 in THP-1 macrophages (Liu et al., 2016), we tested whether BMDM–HG-exo could reproduce this effect. Data shown in Figure 8F demonstrate that BMDM–HG-exo significantly reduce the expression of *Abca1* mRNA in primary BMDM supporting the pro-atherogenic properties of these exosomes when infused into Apoe^−/−^ mice.

Altogether, our observations demonstrate that hyperglycemia dysregulates microRNA levels in myeloid cells and their secreted exosomes and point to miR-486-5p as a candidate EV-associated microRNA that may play an important role in diabetic atherosclerosis acceleration.

## Discussion

Numerous studies recently revealed that EVs contribute to the pathogenesis of diabetes and cardiometabolic diseases, including via their microRNA cargo (Bei et al., 2017; Bouchareychas et al., 2020; Jansen et al., 2017; Krohn et al., 2016). Findings from our laboratory demonstrated that a select set of microRNA carried by macrophage EVs, also termed exosomes, impact the course of atherosclerosis in mice with hyperlipidemia (Bouchareychas et al., 2020). However, their signaling properties in complications of diabetes, including atherosclerosis, remain incompletely understood. In light of the importance of the macrophage in the pathogenesis of atherosclerosis in diabetes, there is a need to elucidate the role of macrophage-derived EVs produced under metabolic stress.

Results of our study reveal a new appreciation for macrophage exosomes in the pathogenesis of diabetic atherosclerosis. Specifically, our findings demonstrate that exosomes produced in a high-glucose environment, simulating human diabetic hyperglycemia levels of 450 mg/dL, induced metabolic reprogramming of recipient cells. Specifically, we noted through bioenergetic studies that naive BMDM cells exposed to BMDM–HG-exo increased glycolytic activity and displayed enhanced proliferation. Furthermore, we observed that when infused into Apoe^−/−^ mice, BMDM–HG-exo increases hematopoiesis and myeloid cell numbers in the circulation that collectively led to larger numbers of macrophages in lesions along with increased apoptotic areas and atherosclerosis progression.

A key observation of this study was a significant increase of CMPs and GMPs in the BM of mice injected with BMDM–HG-exo that was further increased in western diet fed mice. In Apoe^−/−^ mice fed a chow diet, while BMDM-HG-exo were effective in driving hematopoiesis when compared to saline alone, no difference was observed in mice injected with BMDM–HG-exo compared to BMDM–NG-exo showing that western diet feeding could amplify the BMDM–HG-exo effect on CMP/GMP expansion. Numerous studies have reported that the consumption of western diet triggers proliferative hematopoietic cell expansion that is normally regulated by cholesterol efflux mechanisms involving LXRs, ABCA1, ABCG1, and HDL (Wang et al., 2007; Yvan-Charvet et al., 2007, 2010). Our results showed that BMDM–HG-exo decreased *Abca1* mRNA expression in recipient BMDM cells. We could suggest that by decreasing *Abca1*, BMDM–HG-exo further amplified the HSPC expansion induced by the western diet consumption.

Our observations add to findings of several reports showing that diabetes is accompanied by altered hematopoiesis and increased myeloid cell numbers in the circulation (Hazra et al., 2013; Kojima et al., 2014; Nagareddy et al., 2013). So far, this process has been shown to be driven by a systemic increase of S100A8/A9 produced by neutrophils, which signals through the receptor for advanced glycation end products (RAGE) in hematopoietic progenitors cells (Nagareddy et al., 2013) and by upregulating the expression of GLUT-1 on myeloid cells, enhancing glycolytic activity and proliferative signaling resulting in myelopoiesis (Flynn et al., 2020). Although we did not explore this pathway in depth, RNA sequencing analysis of cultured macrophages exposed to BMDM–NG-exo or BMDM–HG-exo showed no differences in levels of RAGE or GLUT-1 gene expression. Surprisingly, we found that only mice fed a chow diet had an increase in hematopoietic progenitors in the spleen after BMDM–HG-exo injections. In those mice, Ly-6C^hi^ monocyte levels were unchanged whereas Ly-6C^low^ monocytes were increased in the same manner as neutrophils. In addition, stimulatory properties of high dietary cholesterol in Western diet fed mice could mask the added effects of the BMDM–HG-exo on further driving myelopoiesis. Alternatively, larger numbers of BMDM–HG-exo may be required to drive hematopoiesis in mice fed a high fat diet as our injection paradigm consisted of only 4.5% of normal circulating exosomes in mice (Huang et al., 2018). Regardless of their exact mode of function and effective concentration dosage, we provide through this study the first evidence that macrophage-derived exosomes produced under high glucose conditions can modulate hematopoiesis and myelopoiesis.

Along with their intrinsic capacity to increase hematopoietic progenitors and circulating leukocytes, we observed that BMDM–HG-exo caused a substantial increase in atherosclerotic lesion area. We noted that such changes included an increase of macrophage foam cell rich area and apoptotic cells in Apoe^−/−^ mice injected with BMDM–HG-exo regardless of dietary challenge. As BMDM–HG-exo were able to increase proliferation in recipient cells, it is plausible that macrophage lesion area was elevated due to an increase in local macrophage proliferation. Indeed, macrophage proliferation locally in lesions has been shown to be a key event in atherosclerosis development (Robbins et al., 2013). Furthermore, results of our recent study of macrophage exosomes in atherosclerosis control demonstrated their ability to infiltrate the aorta of Apoe^−/−^ mice (Bouchareychas et al., 2020).

Support for this line of reasoning comes from reports linking hyperglycemia to altered cell cycle regulation and proliferation (Nagy et al., 2019). While the underlying mechanisms are complex, studies have shown that they involve numerous pathways including AKT/mTOR activation (Lopez et al., 2013), epidermal growth factor (EGF), hypoxia-inducible factor-1/vascular endothelial growth factor-dependent (HIF-1/VEGF) pathways and apoptotic resistance pathway (Li et al., 2019). Such involvement in cellular reprogramming could explain the clinical observations linking high glucose levels with increased prevalence to developing cancer (Li et al., 2019).

In our study, we observed that BMDM–HG-exo could potently induce metabolic reprogramming in recipient cells by failing to increase oxidative phosphorylation, and rather increasing their glycolytic pathway consistent with an entry into the phase S of cell cycle. Importantly, we also observed by sequencing analysis that Homeodomain-interacting protein kinase 2 (HIPK2) was upregulated in BMDM stimulated with BMDM–HG-exo. HIPK2 is a co-regulator of a number of transcription factors and cofactors involved in DNA repair responses, cell proliferation and cell differentiation. Indeed, Hipk2^−/−^ mice show defective hematopoiesis (Aikawa et al., 2006) and HIPK2 depletion by RNA interference has been shown to inhibits cell proliferation in primary BM (Iacovelli et al., 2009). Therefore, our data could support a scenario where BMDM–HG-exo upregulate hematopoiesis and cell proliferation through the HIPK2 signaling pathway that ensure DNA damage repair. Interestingly, we also noted an upregulation of *Bcl2* in BMDM stimulated with BMDM–HG-exo. In addition to its role in regulating apoptosis, BCL-2 has also been found to be a key player in the regulation of mitochondrial function, hematopoiesis, metabolism, autophagy, cell cycle and DNA damage response (Glab et al., 2020). Transgenic mouse models that overexpresses BCL-2 have increased numbers of HSC (Domen et al., 2000) and Ly-6C^low^ monocytes (Bouchareychas et al., 2015) consistent with findings of our study.

Beyond having characterized their cell-signaling properties, we identified several microRNAs that were enriched in BMDM–HG-exo compared to BMDM–NG-exo, including miR-486-5p. Interestingly, we found that PAD + Diabetes-EVs also displayed an increased level of miR-486-5p. In addition, miR-486-5p was enriched in Ly-6C^hi^ monocytes sorted from the Akita mice.

To date, the pathological significance of miR-486-5p has not been fully elucidated and is subject to intense research in cancer biology due to its's role in cell proliferation and cell cycle regulation. Indeed, studies have shown that miR-486-5p is central to the pathogenesis of chronic myeloid leukemia (CML) and aberrant hematopoiesis (Shaham et al., 2015; Wang et al., 2015).

Studies have also shown an increase of miR-486-5p in patients with coronary artery disease (Niculescu et al., 2015). More recently, miR-486-5p enriched in adipose-derived stem cell–EVs have been shown to accelerate the proliferation of skin fibroblasts (Lu et al., 2020). Furthermore, miR-486-5p was shown to reduce ABCA-1 expression in macrophages (Liu et al., 2016) that we reproduced through our studies of BMDM-HG-exo, which in this context could contribute to atherosclerosis acceleration in Apoe^−/−^ mice treated with BMDM–HG-exo, and atherosclerosis in diabetic patients.

Regardless their exact mode of action, our findings uncovered that exosomes produced by macrophages exposed to high glucose concentrations and those isolated from the plasma of diabetic mice and human subjects carry increased levels of select microRNA including miR-486-5p that is associated with dysfunctional hematopoiesis and macrophage cholesterol homeostasis, could explain observations of increased circulating leukocytes and atherosclerosis in diabetic subjects. Further studies will be needed to quantify the proportion of macrophage derived EVs among the pool of EVs in human circulation to further validate whether exosomes produced by macrophages *in vitro* can reflect human physiology.

In summary, our findings reveal a new paradigm to account for experimental and clinical observations of accelerated atherosclerosis in diabetes. Our data show that detrimental properties of hyperglycemia on hematopoiesis can be communicated via exosomes produced by macrophages cultured in a high glucose environment. Mechanisms of action identified in this study include altered energy metabolism and cellular proliferation in target cells. Our data also suggests the possible existence of a feedforward mechanism by which metabolically stressed macrophages can communicate to the BM and spleen via exosomes to accentuate the supply of circulating monocytes and neutrophils in the setting of diabetes. While this process can be seen as detrimental in the context of diabetes, it is plausible that it evolved as a mechanism to help control the need for hematopoietic cell production in response to host infection or need for tissue repair. Our transcriptomics datasets also provide a rich resource for further investigation of the underlying biology of hyperglycemia mediated cellular dysfunction in diabetes. Furthermore, future studies of BMDM exosomes could reveal new opportunities to control cellular dysfunction and cardiovascular complications in diabetes.

### Limitations of the study

Our approach of testing BMDM exosome function *in vivo* includes repeated infusions of an empirical number of BMDM-exosomes that may not accurately reflect physiological levels of exosomes produced by macrophage subtypes in mice. Furthermore, while our studies have identified microRNA cargo associated with exosomes produced by BMDM exposed to high glucose levels and EVs isolated from the plasma of diabetic human subjects, we do not in this study focus on elucidating specific molecular targets of such microRNA. Other possible exosomal cargo including proteins, lipids, and other RNA species that could impact cellular communication were not examined. The co-isolation of minor contaminants including protein aggregates and lipoproteins containing apoAI with EVs may add another level of complexity in the understanding of their function. Future studies will be required to fully understand the contribution of each component of the BMDM–exosomes and their respective targets in contributing to accelerated atherosclerosis in diabetes.

## STAR★Methods

### Key resources table

**Table:** 

REAGENT or RESOURCE	SOURCE	IDENTIFIER
**Antibodies**		

Immunoblotting: Rabbit monoclonal anti-CD9	Abcam	Cat# ab92726; RRID:AB_10561589
Immunoblotting: Mouse monoclonal anti-CD81	Santa Cruz Biotechnology	Cat# sc-166029, RRID:AB_2275892
Immunoblotting: Mouse monoclonal anti-CD63	BD Biosciences	Cat# 556019, RRID:AB_396297
Immunoblotting: Rabbit polyclonal anti-Calnexin	Abcam	Cat# ab10286, RRID:AB_2069009
Immunoblotting: Mouse monoclonal anti-GM130	BD Biosciences	Cat# 610823, RRID:AB_398142
Immunoblotting: Rabbit monoclonal anti-Flotillin-1	Cell Signaling Technology	Cat# 18634, RRID:AB_2773040
Immunoblotting: Mouse monoclonal anti-Alix	Santa Cruz Biotechnology	Cat# sc-53540, RRID:AB_673819
Immunoblotting: Mouse monoclonal anti- Anti- APOA1	Santa Cruz Biotechnology	Cat# sc-376818, RRID:AB_2797313
Immunoblotting: mouse IgG kappa binding protein (m-IgGκ BP)- HRP	Santa Cruz Biotechnology	Cat# sc-516102, RRID:AB_2687626
Immunoblotting: F(ab)2-Goat anti-Rabbit IgG (H+L) Cross-Adsorbed Secondary Antibody, HRP	Thermo Fisher Scientific	Cat# A10547, RRID:AB_2534046
Flow Cytometry: Biotin anti-mouse/human CD45R/B220 antibody	BioLegend	BioLegend Cat# 103204, RRID:AB_312989
Flow Cytometry: Biotin anti-mouse CD4 antibody	BioLegend	Cat# 100508, RRID:AB_312711
Flow Cytometry: Biotin anti-mouse CD8a antibody	BioLegend	Cat# 100704, RRID:AB_312743
Flow Cytometry: Brilliant Violet 421 anti-mouse CD16/32 antibody	BioLegend	Cat# 101332, RRID:AB_2650889
Flow Cytometry: Biotin anti-mouse Ly-6G/Ly-6C (Gr-1) antibody	BioLegend	Cat# 108404, RRID:AB_313369
Flow Cytometry: PE/Cy7 anti-mouse CD150 (SLAM) antibody	BioLegend	Cat# 115914, RRID:AB_439797
Flow Cytometry: Biotin anti-mouse TER-119/Erythroid Cells antibody	BioLegend	Cat# 116204, RRID:AB_313705
Flow Cytometry: Brilliant Violet 510 anti-mouse CD41 antibody	BioLegend	Cat# 133923, RRID:AB_2564013
Flow Cytometry: Biotin anti-mouse CD127 (IL-7Rα) antibody	BioLegend	Cat# 135006, RRID:AB_2126118
Flow Cytometry: PE anti-mouse CD115 (CSF-1R) antibody	BioLegend	Cat# 135505, RRID:AB_1937254
Flow Cytometry: FITC anti-mouse Ly-6C antibody	BioLegend	Cat# 128006, RRID:AB_1186135
Flow Cytometry: PerCP/Cyanine5.5 anti-mouse/human CD11b antibody	BioLegend	Cat# 101228, RRID:AB_893232
Flow Cytometry: APC anti-mouse CD45 antibody	BioLegend	Cat# 103112, RRID:AB_312977
Flow Cytometry: c-Kit Monoclonal Antibody (2B8), APC-Cyanine7	Thermo Fisher Scientific	Cat# A15423, RRID:AB_2534436
Flow Cytometry: CD34 Monoclonal Antibody (RAM34), FITC	Thermo Fisher Scientific	Cat# 11-0341-85, RRID:AB_465022
Flow Cytometry: Ly-6A/E (Sca-1) Monoclonal Antibody (D7), PE	Thermo Fisher Scientific	Cat# 12-5981-83, RRID:AB_466087
Flow Cytometry: CD48 Monoclonal Antibody (HM48-1), APC	Thermo Fisher Scientific	Cat# 17-0481-82, RRID:AB_469408
Flow Cytometry: CD135 (Flt3) Monoclonal Antibody (A2F10), PerCP-eFluor 710	Thermo Fisher Scientific	Cat# 46-1351-82, RRID:AB_10733393
Flow Cytometry: BV786 Streptavidin	BD Biosciences	Cat# 563858, RRID:AB_2869529
TruStain FcX Antibody	BioLegend	Cat# 101320, RRID:AB_1574975
Rat Anti-Mouse Macrophages/Monocytes Monoclonal antibody, Unconjugated, Clone moma-2	Cedarlane	Cat# CL89154, RRID:AB_10086520
Cleaved Caspase-3 (Asp175) Antibody	Cell Signaling Technology	Cat# 9661, RRID:AB_2341188
Donkey anti-Rabbit IgG (H+L) Highly Cross-Adsorbed Secondary Antibody, Alexa Fluor 488	Thermo Fisher Scientific	Cat# A-21206, RRID:AB_2535792
Donkey anti-Rat IgG (H+L) Highly Cross-Adsorbed Secondary Antibody, Alexa Fluor 594	Thermo Fisher Scientific	Cat# A-21209, RRID:AB_2535795

**Chemicals, peptides, and recombinant proteins**		

iScript Reverse Transcription Supermix	Bio-Rad Laboratories	Cat# 1708841
Fast SYBR Green Master Mix	Applied Biosystems	Cat# 4385614
miRCURY LNA RT Kit	Qiagen	Cat# 339340
miRCURY LNA SYBR Green PCR Kit	Qiagen	Cat# 339347
4–20% Mini-PROTEAN TGX Stain-Free Protein Gels	Bio-Rad Laboratories	Cat# 4568094
4x Laemmli Sample Buffer	Bio-Rad Laboratories	Cat# 1610747
Amersham ECL Prime Western Blotting Detection Reagent	GE Healthcare	Cat# RPN2232
Immun-Blot PVDF Membrane	Bio-Rad Laboratories	Cat# 1620177
Penicillin-Streptomycin	Gibco	Cat# 15140122
Dulbecco’s Modified Eagle’s Medium	Corning	Cat# 10-014-CV
RPMI 1640	Corning	Cat# 10-040-CV
Phorbol 12-Myristate 13-Acetate	Fisher Scientific	Cat# BP685-1
Recombinant Murine M-CSF	Peprotech	Cat# 315-02
D-(+)-Glucose solution	Sigma-Aldrich	Cat# G8769-100ML
D-Mannitol	MP Biomedicals	Cat# 02152540-CF
Trypsin-EDTA (0.05%)	Gibco	Cat# 25300054
GlutaMAX	Gibco	Cat# 35050061
RBC Lysis Buffer (10X)	BioLegend	Cat# 420301
CountBright Absolute Counting Beads	Thermo Fisher Scientific	Cat# C36950
RD Western Diet	Research Diets	Cat# D12079B
Teklad Global Diets	Envigo	Cat# 2016
OptiPrep density gradient medium	Sigma-Aldrich	Cat# D1556-250ML
Tissue-Tek O.C.T Compound	Sakura FineTek	Cat# 4583
Oil Red O	Sigma-Aldrich	Cat# O1391
Mayer's Hematoxylin	Thermo Fisher Scientific	Cat# 72804
VECTASHIELD Antifade Mounting Medium with DAPI	Vector Laboratories	Cat# H-1200
Sucrose, 20% Sterile Solution	VWR	Cat# E543-100ML
10X Tris-EDTA, pH 7.4	Fisher Scientific	Cat# BP24771
RNase A/T1 Mix	Thermo Fisher Scientific	Cat# EN0551
Seahorse XF base medium	Agilent	Cat# 103335-100
Seahorse XF 100 mM pyruvate solution	Agilent	Cat# 103578-100
Seahorse XF 200 mM glutamine solution	Agilent	Cat# 103579-100
MethoCult™ GF	StemCell Technologies	Cat# M3434
Streptozocin	Sigma-Aldrich	Cat# S0130
alamarBlue	Bio-Rad Laboratories	Cat# BUF012B
Live Cell Imaging Solution	Thermo Fisher Scientific	Cat# A14291DJ
DiR (DiIC18(7) (1,1'-Dioctadecyl-3,3,3',3'-Tetramethylindotricarbocyanine Iodide))	Invitrogen	Cat# D12731

**Critical commercial assays**		

miRNeasy Mini Kit	Qiagen	Cat# 217004
Quant-iT RiboGreen RNA Assay Kit	Thermo Fisher Scientific	Cat# R11490
Slide-A-Lyzer MINI Dialysis Device, 10K MWCO	Thermo Fisher Scientific	Cat# 88404
Seahorse XFe24 FluxPaks	Agilent	Cat# 102340-100
Seahorse XF Glycolysis Stress Test Kit	Agilent	Cat# 103020-100
Seahorse XF Cell Mito Stress Test Kit	Agilent	Cat# 103015-100
Seahorse XFe24 Cell Culture Microplates	Agilent	Cat# 100777-004
MitoSOX™ Red Mitochondrial Superoxide Indicator	Thermo Fisher Scientific	Cat# M36008
CM-H2DCFDA (General Oxidative Stress Indicator)	Thermo Fisher Scientific	Cat# C6827
CellROX Deep Red Flow Cytometry Assay Kit	Thermo Fisher Scientific	Cat# C10491
FxCycle violet stain	Thermo Fisher Scientific	Cat# F10347
Fixation/Permeabilization Solution Kit	BD Biosciences	Cat# 554714
Qubit Protein Assay Kit	Thermo Fisher Scientific	Cat# Q33211
PKH26 Red Fluorescent cell Linker kit	Sigma-Aldrich	Cat# PKH26GL-1KT
Wako Diagnostics Total Cholesterol E	Fisher Scientific	Cat# 999-02601
Sigma Glucose (GO) Assay Kit	Sigma-Aldrich	Cat# GAGO20-1KT
Fragment Analyzer RNA Kits	Agilent	Cat# DNF-472-0500
Fragment Analyzer DNA/NGS Kits	Agilent	Cat# DNF-474-0500
Universal Plus mRNA-Seq with NuQuant	TECAN	Cat# 0520
MiniSeq High Output Reagent Kit	Illumina	Cat# FC-420-1001
HiSeq 3000/4000 SBS Kit	Illumina	Cat# FC-410-1001
TURBO DNA-free™ Kit	Thermo Fisher Scientific	Cat# AM1907
RNA Clean & Concentrator-5	Zymo Research	Cat# R1016
High Sensitivity RNA ScreenTape	Agilent	Cat# 5067-5579
High Sensitivity RNA ScreenTape Sample Buffer	Agilent	Cat# 5067-5580
SMARTer Stranded Total RNA-Seq Kit v2 - Pico Input Mammalian Components	Takara Bio	Cat# 634418
SMARTer RNA Unique Dual Index Kit	Takara Bio	Cat# 634452
High Sensitivity D1000 ScreenTape	Agilent	Cat# 5067-5584
High Sensitivity D1000 Sample Buffer	Agilent	Cat# 5067-5603
KAPA SYBR FAST Universal qPCR Kit	Kapa Biosystems	Cat# KK4824
NovaSeq 6000 S1 Reagent Kit	Illumina	Cat# 20012864
PhiX Control v3	Illumina	Cat# FC-110-3001
BioAnalyzer High Sensitivity DNA Analysis	Agilent	Cat# 5067-4626
NEXTflex Small RNA Library Prep Kit v3	Perkin Elmer	Cat# NOVA-5132-06

**Deposited data**		

Mouse small RNA -Seq	This paper	GEO Study Accession: GSE162958
Mouse long RNA-Seq	This paper	GEO Study Accession: GSE162961
Human small RNA -Seq	This paper	dbGaP Study Accession: phs002401.v1.p1

**Experimental models: cell lines**		

THP-1	UCSF- Cell and Genome Engineering Core	N/A

**Experimental models: organisms/strains**		

Mouse: B6.129P2-Apoetm1Unc/J	Jackson Laboratories	JAX:002052
Mouse: C57BL/6-Ins2Akita/J	Jackson Laboratories	JAX:003548
Mouse: C57BL6/J	Jackson Laboratories	JAX:000664

**Oligonucleotides**		

Mouse B2m F primer: CTGCTACGTAACACAGTTCCACCC	This paper	N/A
Mouse B2m R primer: CATGATGCTTGATCACATGTCTCG	This paper	N/A
Mouse Gapdh F primer: TGAAGCAGGCATCTGAGGG	This paper	N/A
Mouse Gapdh R primer: CGAAGGTGGAAGAGTGGGAG	This paper	N/A
Mouse Abca1 F primer: ACCTGGAGAGAAGCTTTCAATGA	This paper	N/A
Mouse Abca1 R primer: GTTCAGGTTGACACACTCCATGA	This paper	N/A

**Software and algorithms**		

FlowJo v10.6.2	FlowJo	https://www.flowjo.com/
ImageJ	NIH	https://imagej.nih.gov/ij/
Photoshop CC	Adobe	https://www.adobe.com/products/photoshop.html
Prism 7	GraphPad	https://www.graphpad.com/scientific-software/prism/
2100 Expert Bioanalyzer	Agilent	https://www.agilent.com/en/product/automated-electrophoresis/bioanalyzer-systems/bioanalyzer-software/2100-expert-software-228259
XFe Wave software	Agilent	https://www.agilent.com/en/products/cell-analysis/cell-analysis-software/data-analysis/wave-desktop-2-6
NTA 3.2	Malvern Panalytical	https://www.malvernpanalytical.com/en/support/product-support/software/NanoSight-NTA-software-update-v3-2
NIS Elements BR 4.3	Nikon	https://www.nikon.com/products/microscope-solutions/support/download/software/imgsfw/nis-br_v4300164.htm
ZEN 3.0 Software	Zeiss	https://www.zeiss.com/microscopy/us/products/microscope-software/zen.html
R (version 3.5.0)	R Core Team	https://www.R-project.org/.
STAR aligner software version 2.7.2b	STAR	http://code.google.com/p/rna-star/.

### Resource availability

#### Lead contact

Further information and requests for resources and reagents should be directed to and will be fulfilled by the Lead Contact, Dr. Robert L. Raffai (robert.raffai@ucsf.edu).

#### Materials availability

BMDM-derived exosomes generated in this study will be made available on request, but we may require a payment and/or a completed Materials Transfer Agreement if there is potential for commercial application.

#### Data and code availability

• RNA-seq data have been deposited at GEO accession number: GSE162958 for small RNA and GEO: GSE162961 for long RNA and human data are accessible through the accession number dbGaP: 682 phs002401.v1.p1 and are publicly available as of the date of publication. Accession numbers are listed in the key resources table.
• This paper does not report original code.
• Any additional information required to reanalyze the data reported in this paper is available from the lead contact upon request.

### Experimental model and subject details

#### Animals

All animal experiments were approved by the Institutional Animal Care and Use Committee at the VA Medical Center. Approximately 8 to 10-week-old male C57BL/6J (000664) wild type (WT), 8 to 10-week-old male C57BL/6-Ins2Akita/J (003548) and 25 to 30-week-old male Apoe ^tm1Unc^/J (Apoe^−/−^) mice on C57BL/6 background were used in this study. All mice were obtained from The Jackson Laboratory (Sacramento, CA), bred and housed in specific pathogen–free conditions in the Animal Research Facility at the San Francisco VA Medical Center.

To induce diabetes, 10-week-old WT mice were given daily intraperitoneal injections of STZ (50 mg/kg body weight) dissolved in 0.05 M sodium citrate buffer for 5 days. Controls were injected with an equivalent volume of sodium citrate buffer. Diabetes induction was monitored by blood glucose measurements sampled from the tail vein (accu-Check).

Because wild-type mice do not spontaneously develop atherosclerosis, we choose the Apoe^−/−^ mouse model which is the most widely used pre-clinical model of atherosclerosis to study the effect of exosomes produced in high glucose *in vivo*.

The use of western diet feeding is recognized to drive the process of hematopoiesis in this model. Therefore, we choose to use both chow and western fed Apoe^−/−^ mouse model to distinguish the BMDM-exo effect from the priming effect of the western diet on HSPC expansion. Ten-week-old male Apoe^−/−^ mice were fed a Western diet (Research Diets) for 10 weeks or 26-week old male Apoe^−/−^ fed a chow diet were used for hematopoiesis and atherosclerosis assessment.

In this study, exosomes were infused intraperitoneally (IP) as we previously reported that IP-infused exosomes could target hematopoietic compartments as well as aorta (Bouchareychas et al., 2020). Exosomes were infused three times a week for 4 weeks with saline (PBS) or with 1x10^10^ BMDM-derived exosomes. Mice that appeared unhealthy (body weight <2 SD below average) or with skin lesions were excluded.

#### Primary cells used in this study

Bone marrow-derived macrophage (BMDM) and hematopoietic stem cells (HSC) were derived from male C57BL/6J mice ranging from 8 to 12 weeks of age.

### Method details

#### Cell culture

Murine BMDM were obtained as described previously (Bouchareychas et al., 2017). Briefly, BM cells were flushed from the tibia and femurs of 8-week-old WT C57BL/6 mice. Cells were cultured in Dulbecco’s Modified Eagle’s Medium (Corning) supplemented with 10% fetal bovine serum (GIBCO), 1% GlutaMax (GIBCO), and 1% penicillin-streptomycin (GIBCO) and differentiated with 25 ng/ml mouse M-CSF (Peprotech) for 6 days. For exosome isolation, BMDM were cultured in exosome-free media (EFM) prepared by ultracentrifugation for 18 h at 100,000 x g (Type 45 Ti rotor, Beckman Coulter) and filtration (0.2 mm). BMDM were washed two times with PBS and cultured for 24 h with 19.5mM glucose (Sigma-Aldrich) to produce BMDM–HG-exo or 19.5mM Mannitol (MP Biomedicals) for BMDM–NG-exo. For *in vitro* experiments, BMDM were dispensed into 12-well culture plates (Corning) at a concentration of 0.3 x10^6^ cells/well and stimulated with BMDM–NG-exo or BMDM–HG-exo for 24 h at a concentration of 2x10^9^ particles per ml. The THP-1 human monocytic cell line was maintained in RPMI 1640 media supplemented with 10% FBS and 1% penicillin-streptomycin (GIBCO) and differentiated into macrophages using a treatment with phorbol-12-myristate acetate (50 ng/ml) for 48h followed by 24 h incubation in RPMI complete medium.

For CFUs assay, 2 x10^4^ total BM cells isolated from the legs and hips of 8-week-old male WT mice were cultured in Methylcellulose-based medium with a recombinant cytokine mix (StemCell Technologies). Cells were treated with PBS, BMDM–NG-exo or BMDM–HG-exo every two days at a dose of 2 x10^9^ particles/ml. Number of the colonies were quantified after 12 days of culture using a Zeiss Axio Observer microscope and ZEN 3.0 Software.

#### EVs isolation and nanoparticle tracking analysis

EVs were isolated from conditioned cell culture medium or from 1ml of human plasma by Cushioned-Density Gradient Ultracentrifugation (C-DGUC) as previously described (Bouchareychas et al., 2020). We used the terminology EVs to refer to human exosomes as there is more vesicle heterogeneity in particle sizes and contamination for other vesicular structures in human EVs as compared to EVs isolated from cultured cells.

To produce BMDM exosomes, BM cells were plated onto a 150 mm round dishes at a density of 5x10^6^ cells per dish. Cell culture supernatant (20 ml) was harvested from an average of 20 plates with 5x10^6^ cells/plate and an average viability of 96% for BMDM. The collected media were subjected to 400 x g centrifugation for 10 min at 4°C followed by 2000 x g centrifugation for 20 min at 4°C and filtered (0.2 mm).

Human plasma was first centrifuged at 1,500 x g for 10 minutes at 4°C and 1 ml was diluted in 38.5 mL of cold PBS. Cell culture supernatant or diluted plasma was centrifuged on a 60% iodixanol cushion (Sigma-Aldrich) at 100,000 x g for 3 h (Type 45 Ti or Type 50.2 Ti rotor, Beckman Coulter). The bottom 3 mL was then collected and underlaid below a step density gradient composed of three 3 ml layers (5%, 10%, 20% w/v OptiPrep iodixanol) in a Beckman Coulter Ultra-Clear centrifuge tube. The density gradient was subsequently spun at 100,000 x g for 18 h at 4°C (SW 40 Ti rotor, Beckman Coulter). Subsequently, twelve 1 mL fractions were collected starting from the top of the tube. Fractions 7 and 8 of the gradient were dialyzed in PBS with the Slide-A-Lyzer MINI Dialysis Device (Thermo Fisher Scientific) and used for subsequent experiments and analyses. NanoSight LM14 (Malvern Instruments, Westborough, MA) was used to measure EVs size and concentration using a 488-nm detection wavelength. The analysis settings were optimized and kept identical for each sample, with a detection threshold set at 3, three videos of 1min each were analyzed to give the mean, mode, median, and estimated concentration for each particle size. Samples were diluted in 1:100 or 1:200 PBS and measured in triplicates. Data were analyzed with the NTA 3.2 software. All the EVs samples were stored at 4°C and used within one month after their isolation.

#### Immunoblotting

For western blot analysis, an equal volume of the EV fractions were mixed with Laemmli buffer (Bio-Rad Laboratories) and boiled at 95°C for 5 minutes. Samples were then loaded on a 10% SDS-PAGE gel and transferred onto PVDF membrane (Bio-Rad Laboratories). The membranes were blocked with 5% non-fat milk dissolved in PBS for 1 h and then probed with primary antibodies overnight at 4°C (primary antibodies: anti-CD9 (1:100, Abcam), anti-Flotillin (1:500, Cell Signaling), anti-Alix (1:100, Santa Cruz Biotechnology), anti-Calnexin (1:500, Abcam), anti-GM130 (1:250, BD Biosciences), anti-CD81 (1:100, Santa Cruz Biotechnology), anti-CD63 (1:100, BD Pharmingen), anti-APOA1 (1:500, Santa Cruz Biotechnology). After 4 washes in PBS containing 0.1% Tween (PBST), membranes were incubated with corresponding HRP-conjugated secondary antibodies: anti-mouse IgG-HRP (1:1000, Santa Cruz Biotechnology), anti-rabbit IgG-HRP (1:1000, Thermo Fisher Scientific) or anti-rat IgG-HRP (1:1000, Thermo Fisher Scientific) for 1h and washed in PBST. Signals were visualized after incubation with Amersham ECL Prime substrate and imaged using an ImageQuant LAS 4000.

#### Transmission electron microscopy

An assessment of exosome morphology was assessed by Electron microscopy by loading 7x10^8^ particles onto a glow discharged 400 mesh Formvar-coated copper grid (Electron Microscopy Sciences). The nanoparticles were left to settle for two minutes, and the grids were washed four times with 1% Uranyl acetate. Excess Uranyl acetate was blotted off with filter paper. Grids were then allowed to dry and subsequently imaged at 120kV using a Tecnai 12 Transmission Electron Microscope (FEI).

#### Exosomes labeling

Fluorescently labelled BMDM-derived exosomes were generated using PKH26 (Sigma-Aldrich) or with DiR (DiIC_18_(7) (1,1'-Dioctadecyl-3,3,3',3'Tetramethylindotricarbocyanine Iodide) (Invitrogen) according to the manufacturer’s instructions. Briefly, PKH26 or DiR dye was added to the 3mL iodixanol cushion layer containing exosome or to 3ml of PBS to achieve a final concentration of 3.5 μM for PKH26 and 1 μM for DiR and incubated for 5 min and 20 min respectively at room temperature. Next, we loaded the labeled cushion layer below an iodixanol step gradient as described in the EVs isolation section. Free dye and non-specific protein-associated dye was eliminated from labeled exosomes or from PBS control during this separation step. BMDM cells were then incubated with 2x10^9^ PKH26-labeled exosomes for 2 h, washed 3 times with PBS and imaged using a Nikon Eclipse Ni microscope and data were analyzed using ImageJ.

Three groups of 25 weeks old male Apoe^−/−^ mouse of similar weight and fed a chow diet were injected with PBS or 1×10^10^ BMDM–NG-DiR exosomes, either intravenously (IV) via the retro-orbital plexus or intraperitoneally (I.P). Subsequently, a similar blood volume was collected from each mouse at the 2, 4-, 8- and 24-h timepoints post-injection and IR intensity was detected using the Odyssey Infrared Imaging System and Image Studio software.

#### RNA extraction, whole transcriptome library preparation, and Sequencing

Exosomes preparations were treated with 0.4 mg/ml of RNase A/T1 Mix (Thermo Fisher Scientific) for 20 min at 37°C before RNA extraction. Total RNA isolated from cells and exosomes was purified using the miRNeasy Mini Kit (Qiagen) according to the manufacturer’s protocol. RNA was quantified using Nanodrop or Quant-iT RiboGreen RNA Assay Kit (Thermo Fisher Scientific) and reverse transcribed using the iScript Reverse Transcription Supermix (Bio-Rad Laboratories) for mRNA or the miRCURY LNA RT Kit (Qiagen) for microRNA analysis. PCR reactions were performed using the Fast SYBR Green Master Mix (Applied Biosystems) for mRNA or the miRCURY LNA SYBR Green PCR Kit (Qiagen) for microRNA and run on a QuantStudio 7 Flex Real-Time PCR System. Ct values were normalized to the housekeeping gene Gapdh and B2m for mouse mRNA. For microRNA expression, UniSp6 was used as a spike-in control (Qiagen). The following real time primers were used: 5’-ACCTGGAGAGAAGCTTTCAATGA-3’ (forward) and 5’-GTTCAGGTTGACACACTCCATGA-3’ (reverse) for mouse ABCA1; 5’-CTGCTACGTAACACAGTTCCACCC-3’ (forward) and 5’-CATGATGCTTGATCACATGTCTCG-3’ (reverse) for mouse B2m; 5’-TGAAGCAGGCATCTGAGGG-3’ (forward) and 5’-CGAAGGTGGAAGAGTGGGAG-3’ (reverse) for mouse Gapdh. primers for miR-486-5p were purchased from Qiagen.

For sequencing analysis, isolated RNA sample was DNase treated with TURBO DNA-free (Thermo Fisher), then purified and concentrated with Zymo RNA Clean & Concentrator – 5 (Zymo Research). The RNA was measured for quantity with Quant-iT Ribogreen RNA Assay (Thermo Fisher) and quality with Agilent High Sensitivity RNA Screen Tape and buffer (Agilent). For mouse RNA samples, an indexed, Illumina-compatible, double-stranded cDNA whole transcriptome library was synthesized from 10ng of total RNA with Takara Bio’s SMARTer Stranded Total RNA-Seq kit v2 Pico Input Mammalian (Takara Bio) and SMARTer RNA Unique Dual Index Kit (Takara Bio). Library preparation included RNA fragmentation (94°C for 4 min), cDNA synthesis, a 5-cycle indexing PCR, ribosomal cDNA depletion, and a 12-cycle enrichment PCR. Each library was measured for size with Agilent’s High Sensitivity D1000 ScreenTape and reagents (Agilent) and concentration with KAPA SYBR FAST Universal qPCR Kit (Kapa Biosystems). Libraries were then combined into an equimolar pool which was also measured for size and concentration. The pool was clustered onto a flowcell (Illumina) with a 1% v/v PhiX Control v3 spike-in (Illumina) and sequenced on Illumina’s NovaSeq 6000 at a final flowcell concentration of 400pM. The first and second reads were each 100 bases.

For human RNA samples, the integrity of total RNA was verified on Fragment Analyzer (Agilent), and only RNA with RQN number of above 8 was used for library constructions. A starting quantity of 70 ng of total RNA was used according to vendor instructions with Universal plus mRNA with NuQuant (TECAN), final library PCR amplification was 16 cycles. After library completion, individual libraries were pooled equally by volume, quantified on Fragment Analyzer (Agilent). Quantified library pool was diluted to 1nM and sequenced on MiniSeq (Illumina) to check for quality of reads. Finally, individual libraries were normalized according to Illumina's MiniSeq output reads, specifically by % protein coding genes. The library pool was clustered onto 4 lanes of Illumina's HiSeq4000 single end read (SE) 50 (Illumina).

#### NEXTFLEXSmall RNALibrary Preparation

Small RNA libraries were generated using NEXTflex Small RNA Library Prep Kit v3 (PerkinElmer) following the manufacturer’s instructions using gel purification without adapter inactivation and 16 cycles of PCR amplification. Following PCR amplification, libraries between 140 and 160bp in size were gel purified using 6% TBE gels followed by ethanol precipitation and resuspension in 20ul of ultra-pure water.

#### RNA-sequencing data analysis

The raw sequence image files from the Illumina HiSeq in the form of bcl files were converted to the fastq format using bcltofastq v.2.19.1.403 and checked for quality to ensure the quality scores did not deteriorate at the read ends.

For small RNA data processing, the Bioo Scientific NEXTflexadapters and random 4bp ends were clipped from the reads using cutadapt v.1.14. Reads shorter than 15 nts were discarded and after adaptor trimming, the 30 bases below a quality score of 30 were also trimmed. The reads are first mapped to a library of UniVec contaminants, a collection of common vector, adaptor, linker and PCR primer sequences collated by the NCBI. They are then mapped to mouse rRNA sequences obtained from NCBI. Finally, small RNA mapping and annotation was done using sRNABench (v.3-3.2) where reads are mapped to the mouse genome (GRCm38) and transcriptome using a transcriptome which contains all ensembl genes plus annotations for microRNAs, as obtained for miRBase (v.22). Alignment files are then processed by sRNABench into miRNA counts matrices for further analysis.

For mouse whole transcriptome data processing, the SMARTer Total RNA pico v2 reads are quality filtered and trimmed as recommended by Takara Bio with the removal of the first 3 bases of read2. After trimming and filtering reads are genome and transcriptome mapped using STAR (v. 2.5.3a). Aligned BAM files are converted into gene counts matrices for further analysis using FeatureCounts (v.2.0.1), using read2 as the sense strand. For RNAseq analysis, differential expression was conducted using the DESeq2 package (version 1.20.0) in R (version 3.5.0) for all gene expression analyses. The raw read counts for the samples were normalized using the median ratio method (default in DESeq2). The significant differentially expressed genes (by Benjamini-Hochberg adjusted p values) are reported in the paper. Heatmaps were created using the pheatmap (v.1.0.10) package in R.

GO analyses were performed using PANTHER GO-slim Biological Process and DAVID with a FDR threshold at ≤ 0.05 or <0.01 as indicated on each figure.

For human whole sequencing analysis, reads were aligned to the human genome GRCh38 and quantified using the STAR aligner software version 2.7.2b. Differential expression analysis was performed in the R computing environment version 3.6.1 using the software DESeq2 version 1.26. FDR-corrected p-values were used to evaluate significant differences between experimental groups using a significance threshold of 0.05. A minimum differential expression of log2 fold change +/- 1 was also used to determine significance. Gene ontology analyses were conducted using PANTHER GO-slim Biological Process.

#### Primary cell preparation and purification

Leukocytes subsets and hematopoietic stem and progenitors cells (HSPC) were identified as previously described (Bouchareychas et al., 2020). Briefly, blood was drawn by retro-orbital puncture into EDTA-coated tubes. Erythrocytes were lysed in RBC lysis buffer (BioLegend). Samples were then centrifuged at 300 x g and 4°C and incubated with TruStain FcX (BioLegend) for 10 min at 4°C in FACS buffer before staining with appropriate Abs: CD11b (clone M1/70), Ly-6C (clone HK1.4), CD115 (clone AFS98) CD45 (clone 30-F11) (all BioLegend) for 30 min at 4°C. The antibody dilutions ranged from 1:200 to 1:100. For microRNA sequencing analysis, a total of 50,000 Ly-6C^hi^ monocytes were sorted from the blood of diabetic WT mice treated with STZ or Akita mice and their respective non-diabetic controls using a FACSAria IIIU cell sorter (BD Biosciences). Peritoneal cells were collected by lavage with 10 ml cold PBS using a 16-G needle. Cells were then plated in 6-well plates and harvested 6 h later.

For HSPC staining, murine femora were excised, flushed with 10 ml of sterile PBS and filtered through 40 μm filters. Cells were centrifuged at 300 x g and 4°C for 5 min and the red blood cells were lysed. Cells were washed, re-centrifuged and stained in FACS buffer containing a lineage-marker cocktail of biotinylated anti-CD4 (RM4-5), -CD8 (53-6.7), -B220/CD45RA (RA3-6B2), -TER-119 (TER-119), -Gr-1 (RB6-8C5), and -CD127 (IL-7Ra/A7R34) antibodies (all from BioLegend). These cells were then stained with anti-CD34 (RAM34, eBioscience), anti-CD150 (TC15-12F12.2, BioLegend), anti-CD48 (HM48-1, Invitrogen), anti-Sca-1 (D7, Invitrogen), anti-CD135 (A2F10, Invitrogen), anti-c-Kit (2B8, Life Technologies), anti-CD16/32 (93, BioLegend), anti-CD41 (MWReg30, BioLegend) and streptavidin-BV786 (BD Biosciences). Sca1+ c-Kit+ (LSK) Flt3– CD34– was used as HSC-gating parameters. MPP1 population was defined as CD34+ LSK CD150+ CD48– cells, MPP2 as CD34+ LSK CD150+ CD48+ cells, MPP3 as CD34+ LSK CD150– CD48+ cells and MPP4 as CD34+ LSK Flt3+ CD150– CD48+ cells. Common myeloid progenitors (CMPs) were gated as Sca-1– c-Kit+ CD41– CD34+ CD16/32–. The granulocyte/macrophage progenitors (GMPs) was defined as Sca-1– c-Kit+ CD41– CD34+ CD16/32+ and megakaryocyte/erythrocyte progenitors (MEPs) as Sca-1– c-Kit+ CD41– CD34– CD16/32–. Doublets, dead cells, and Lin– cells were excluded prior gating analysis. Absolute numbers of cells were calculated using CountBright Absolute Counting Beads (Thermo Fisher Scientific). Data were acquired on an LSRII flow cytometer (BD Biosciences) and analyzed with FlowJo v10.6.2 (FlowJo).

#### Cholesterol

Plasma total cholesterol was quantified using the Wako Total E cholesterol kit following the manufacturer’s instructions.

#### Atherosclerotic lesion analysis

Aortic root sections were stained and quantified as previously described (Bouchareychas et al., 2015). Hearts were excised following perfusion with PBS, fixed in 10% formalin, incubated overnight in 20% sucrose and embedded in OCT. For Apoe^−/−^ mice, hearts were sectioned with a cryostat (10 μm), stained with oil red O (Sigma-Aldrich) and counterstained with modified Mayer’s hematoxylin (Thermo Fisher Scientific). Lesion area was quantified by averaging six sections that were spaced 50 μm apart, starting from the base of the aortic root. Necrotic core area was measured by determining the acellular region (DAPI negative) in each plaque. For macrophage staining, sections were labeled with a primary rat anti-mouse MOMA-2 antibody (Cedarlane labs) and detected with an AlexaFluor 594 donkey anti-rat IgG antibody (Thermo Fisher Scientific). To assess apoptotic cells in lesion, sections were stained for cleaved caspase-3 (Cell Signaling, 1/200) staining followed by AlexaFluor 488 donkey anti-rabbit IgG (Thermo Fisher Scientific). Total cleaved caspase 3 positive area was calculated and divided by the total atherosclerotic plaque area measured by ORO in serial sections. Images were captured with a Nikon Eclipse Ni and a Zeiss Observer microscope, analyzed using NIS-Elements and ZEN 3.0 Software. Analyses were performed in a blinded manner.

#### Seahorse extracellular flux analysis

BMDM were plated at 60,000 cells/well into XFe24 cell culture microplates (Agilent) and incubated overnight at 37°C and 5% CO_2_. The following day, BMDM were stimulated with PBS, BMDM–NG-exo or BMDM–HG-exo for 24 h. For the cell Mito stress test, cells were washed with Seahorse XF base medium (Agilent) supplemented with 10 mM glucose (Sigma-Aldrich), 1 mM pyruvate (Agilent) and 2 mM glutamine (GIBCO) and incubated for 1 h at 37°C without CO_2_. For the Glycolysis stress test, cells were placed in Seahorse XF base medium supplemented with 2 mM glutamine. OCR and ECAR were measured using the mitochondrial stress test kit (Agilent) and Glycolysis stress kit with the Seahorse XFe-24 Bioanalyzer (Agilent) and were analyzed using XFe Wave software. All OCR/ECAR measurements were normalized to cell count.

#### Glucose and ROS measurements

Glucose was quantified in the culture supernatant of the BMDM exposed to 5.5mM or 25mM glucose using the Glucose (GO) Assay Kit (Sigma-Aldrich) following the manufacturer’s instructions.

BMDM were plated in 96 well plates and treated with elevated glucose levels (25 mM glucose) or osmotic normal glucose levels (5.5 mM glucose +19.5 mM mannitol) for 24 h. Culture medium was then removed, cells washed with PBS, then incubated with MitoSOX (5μM final, Invitrogen) or CM-H2DCFDA (10μM, Invitrogen) in Live Cell Imaging Solution (Invitrogen) for 30 min at 37°C. Cells were washed twice and the fluorescence signal was measured in a microplate reader (CM-H2DCFDA 488/543 nm, MitoSOX 510/580 nm). For CellROX analysis, BMDM were plated in 12-well plates at 0.3 x10^6^ cells/ml with 5.5mM or 25mM glucose for 24 h. Thirty minutes before the end of the stimulation, CellROX (5 μM) was added directly into the cell culture medium and incubated at 37°C for 30 min. Cells were washed, removed from plates with cellstripper (Corning), pelleted at 300 x g for 5 min, resuspended and subjected to FACS analysis using CytoFlex S cytometer (Beckman-Coulter).

#### Cell proliferation

The proliferation of cultured BMDM was measured at 4 h and 24 h after exosome stimulation using the alamarBlue assay (Bio-Rad Laboratories). BMDM were plated in 96 well plates and treated with PBS, BMDM–NG-exo or BMDM–HG-exo for 24 h with 10% (vol/vol) AlamarBlue in culture medium and incubated at 37°C, 5% CO2. Fluorescence intensity was read using a microplate spectrofluorometer at excitation 560 nm and emission 590 nm. Cell-cycle analysis was performed using FxCycle violet stain (Invitrogen). BMDM were plated in 12 well plates at 0.3 x10^6^ cells/ml and treated with PBS, BMDM–NG-exo or BMDM–HG-exo (2x10^9^ particle/ml) for 4 h. Cells were washed with warmed PBS, removed from plates with cellstripper (Corning), pelleted at 300 x g for 5 min, resuspended in fixation/permeabilization solution (BD Bioscience) for 15 min. Cells were washed with Perm/Wash Buffer (BD Bioscience), centrifuged at 300 x g for 5 min and resuspended in Perm/Wash Buffer containing FxCycle violet stain, incubated for 30 min and subjected to FACS analysis.

### Quantification and statistical analysis

Statistical analysis was performed using GraphPad Prism v8.4.3, and the tests used were unpaired, two-tailed, Student’s t test (two groups) and one-way analysis of variance (ANOVA) with post-tests as indicated in figure legends for multiple groups. ∗p <0.05, ∗∗p < 0.01, ∗∗∗p < 0.001 ∗∗∗∗p < 0.0001. All error bars represent the mean ± the standard error of the mean (SEM unless stated). All experiments were repeated at least twice or performed with independent samples.
