# Supplementary material for: High glucose macrophage exosomes enhance atherosclerosis by driving cellular proliferation & hematopoiesis
Source: iScience. 2021 Jul 10;24(8):102847. doi: 10.1016/j.isci.2021.102847 (PMC8333149; doi:10.1016/j.isci.2021.102847)
Supplement: Document S1. Figures S1–S3 [file mmc1.pdf]

iScience, Volume *24*

Supplemental information

High glucose macrophage exosomes enhance atherosclerosis by driving cellular proliferation & hematopoiesis

Laura Bouchareychas, Phat Duong, Tuan Anh Phu, Eric Alsop, Bessie Meechoovet, Rebecca Reiman, Martin Ng, Ryo Yamamoto, Hiromitsu Nakauchi, Warren J. Gasper, Kendall Van Keuren-Jensen, and Robert L. Raffai

Figure S1

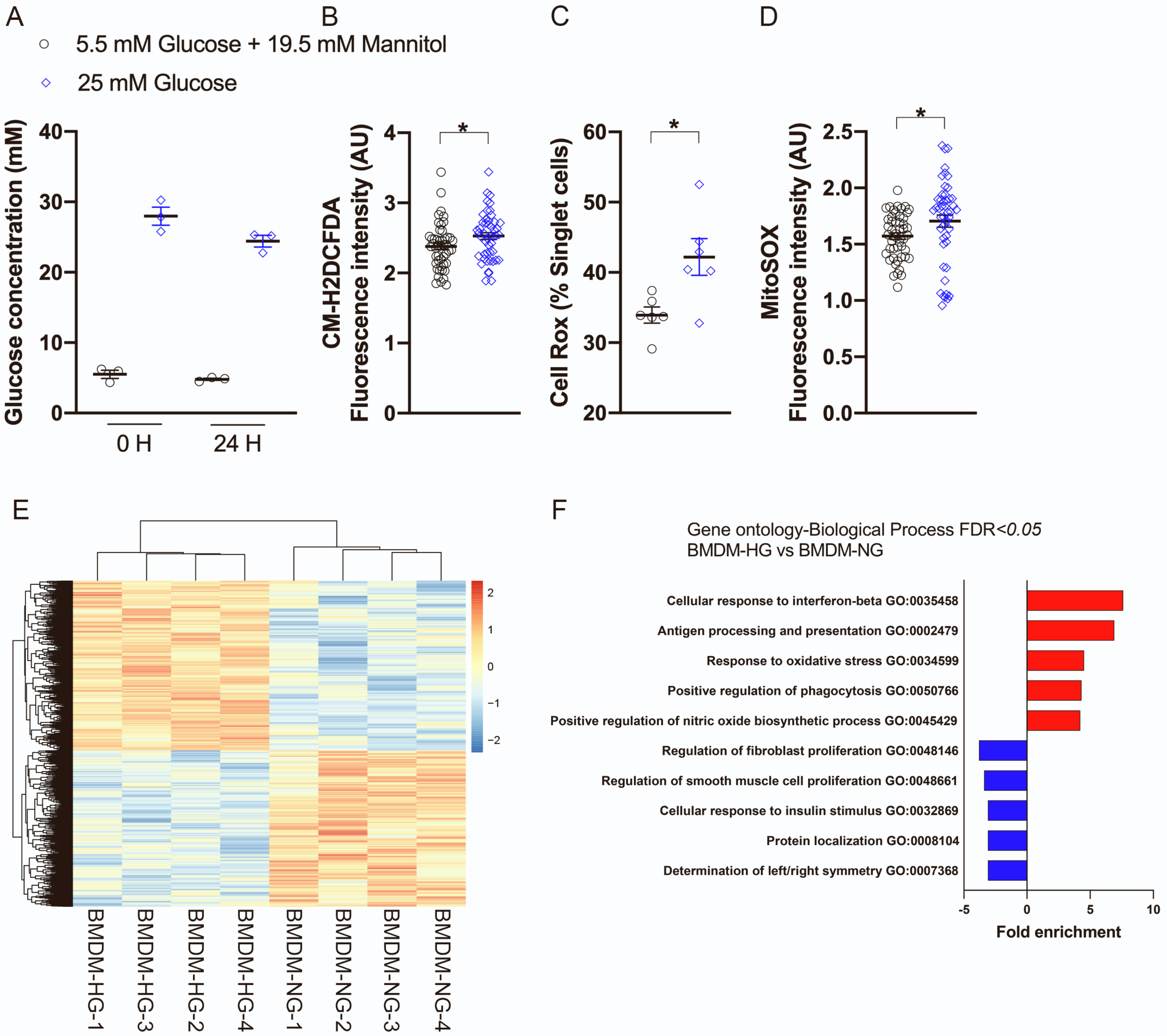

Figure S1. Related to Figure 1. Effect of high glucose concentration on BMDM.
(A) Glucose measurement in BMDM culture supernatant at t=0 and after 24h of culture. (B) Measurement of CM-H2DCFDA fluorescence intensity in BMDM culture in 25 mM glucose concentration or normoglycemic conditions (5.5 mM glucose +19.5 mM mannitol) for 24 hours. (C) Flow cytometry analysis of reactive oxygen species using CellRox. One representative experiment out of two experiments is shown n=6 mice per group.
(D) Measurement of MitoSOX fluorescence intensity in BMDM culture in 25 mM glucose concentration or normoglycemic conditions (5.5 mM glucose+19.5 mM mannitol) for 24 hours.
(E) Heat map showing the distinct mRNA expression profiles between BMDM culture in 25 mM (BMDM-HG, n = 4) or 5.5 mM glucose concentration (BMDM-NG, n = 4).
(F) GO enrichment analysis (Biological process) of the gene differential expressed in BMDM culture in 25 mM (n = 4) or 5.5 mM glucose concentration. The minimum count of genes considered for the analysis was >10.
*p < 0.05; **p < 0.01 as determined by unpaired Student's t test analysis. Data are represented as mean ± SEM.

Figure S2

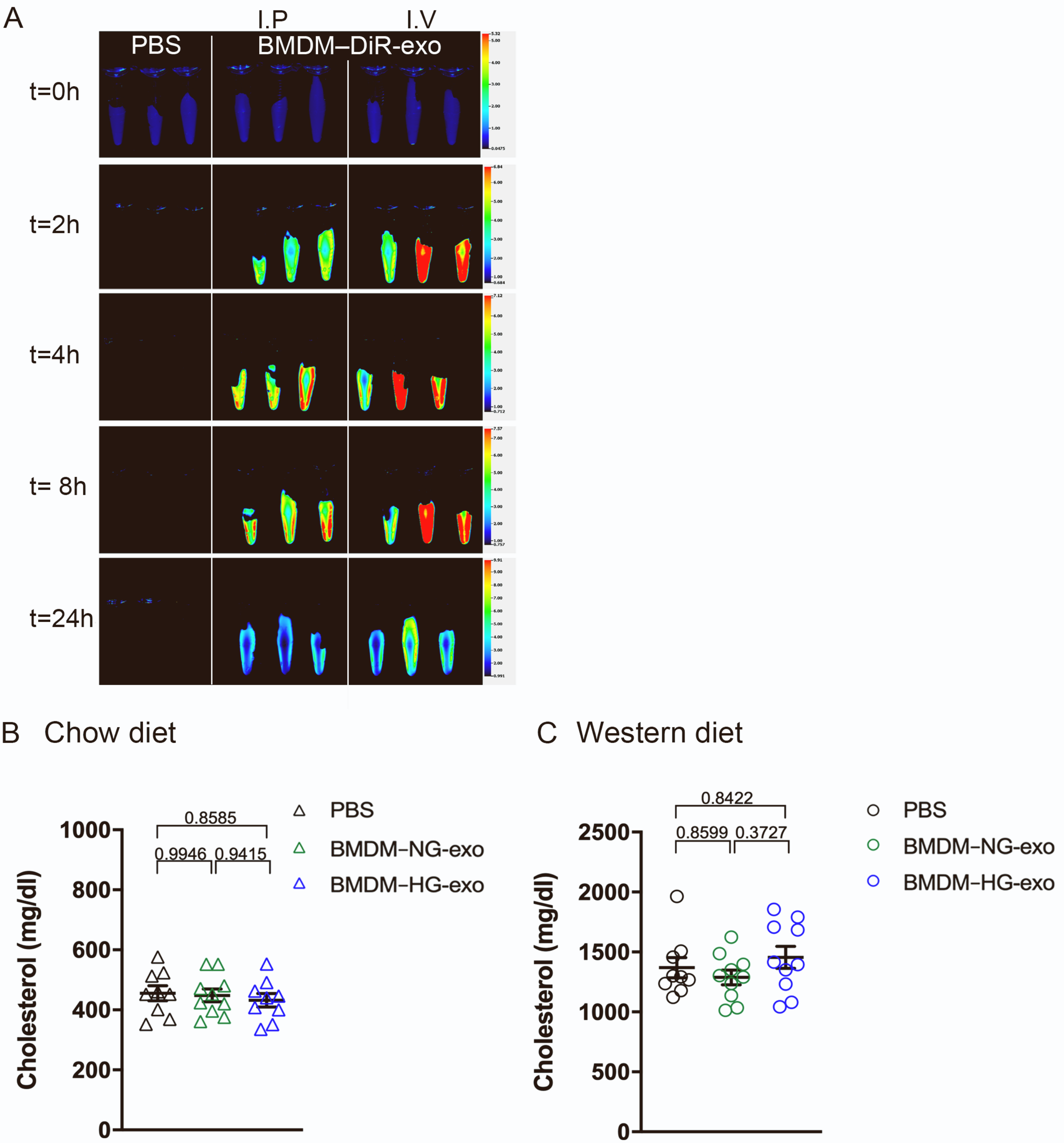

Figure S2. Related to Figure 2. Kinetics of IP versus IV injection routes for DiR-labelled EVs delivery to the bloodstream and cholesterol measurements.

(A) Three groups of 25 weeks-old male Apoe$^{-/-}$ mice of similar weight and fed a chow diet were injected with 1x 10^{10} BMDM–NG-DiR exosomes, or PBS alone either IV via the retro-orbital plexus or I.P. Subsequently, a similar blood volume was collected from each mouse at the 2, 4, 8 and 24 hour time points post-injection and IR intensity was detected on an Odyssey instrument.

Plasma cholesterol levels from Apoe$^{-/-}$ mice fed a chow diet (B) or Western diet (C) after 4 weeks of injections with PBS, BMDM–NG-exo or BMDM–HG-exo. Statistical analysis was performed using a two-way ANOVA with Sidak’s multiple comparisons post-test. *$p < 0.05$. Data are represented as mean ± SEM.

Figure S3

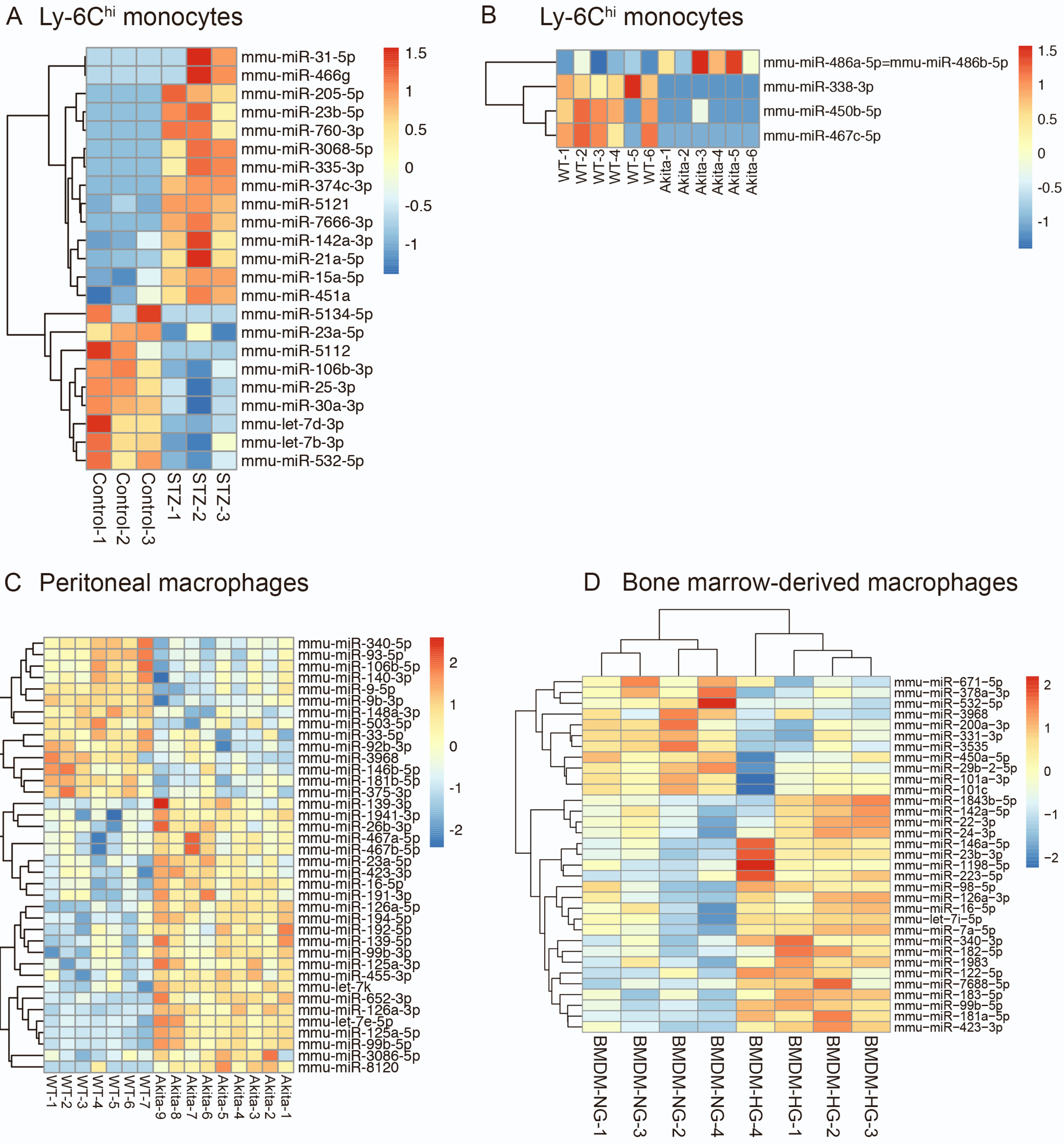

Figure S3. Related to Figure 8. Hyperglycemia induces microRNA dysregulation.
(A) Heat map showing the distinct microRNA expression profiles in Ly-6Chi monocytes isolated from STZ mice and (B) Akita mice (padj <0.05). (C) Heat map illustrating microRNAs differential expression in peritoneal macrophage isolated from Akita and controls mice. Red signal and blue signal indicate microRNA expression levels (padj <0.05). (D) Heat map illustrating microRNAs differential expression in BMDM culture in 25mM glucose concentration (BMDM-HG) or normoglycemic conditions (5.5mM glucose+19.5mM mannitol, BMDM-NG) for 24 hours (p <0.05).
